# NLRP1 inflammasome activation in skin equivalents reveals mechanistic insights into the roles of keratinocytes in psoriasis

**DOI:** 10.1038/s41419-026-08908-6

**Published:** 2026-05-30

**Authors:** Michela Di Filippo, Tugay Karakaya, Marta Slaufova, Phil F. Cheng, Paulina Hennig, Petra Boukamp, Steve Pascolo, Julia-Tatjana Maul, Isabel Kolm, Mitchell P. Levesque, Thomas Kündig, Hans-Dietmar Beer

**Affiliations:** 1 Department of Dermatology, University Hospital Zurich, Zurich, Switzerland; 2 DKFZ, Heidelberg, Germany; 3 Faculty of Medicine, University of Zurich, Zurich, Switzerland

**Keywords:** Psoriasis, Inflammasome

## Abstract

The pro-inflammatory cytokines interleukin(IL)-1β and IL-36γ are key drivers of psoriasis, an inflammatory skin disease for which a causal therapy is not available. However, the mechanisms underlying regulation of these cytokines in psoriasis remain poorly understood. Generation of IL-1β activity is regulated by inflammasomes. We activated the NLRP1 inflammasome in human keratinocytes cultivated in three-dimensional skin equivalents. NLRP1 activation induced histological and molecular features highly reminiscent of psoriasis. Mechanistically, the phenotype was dependent on IL-1, which triggered a pro-inflammatory epidermal-dermal crosstalk. This included induction of IL-36γ expression, which was released from keratinocytes through NLRP1 inflammasome-induced gasdermin D pores. The relevance of these findings is reflected by the expression of NLRP1 and inflammasome activation in lesions of psoriasis patients. Finally, we discovered endogenous cytoplasmic double stranded (ds) RNA, recently associated with cellular perturbations in psoriasis, as a novel NLRP1 activator. Our results identify a novel endogenous dsRNA-mediated NLRP1-IL-1-IL-36γ signaling axis relevant in psoriasis and suggest its targeting as a promising treatment strategy.

## Introduction

Psoriasis is a chronic inflammatory disease affecting 2–3% of the population, characterized by scaly skin lesions T cells infiltration, and epidermal thickening [1, 2]. Although primarily cutaneous, it is increasingly recognized as a systemic disorder [3]. Psoriasis is genetically complex, with over 80 risk loci associated and multiple clinical forms [3, 4]. Psoriasis vulgaris (PV), the most common form, is driven mainly by IL-23 and IL-17 pathways [1], whereas generalized pustular psoriasis (GPP), a rare but live-threatening form, is frequently caused by loss-of-function mutations in the IL-36 receptor antagonist (IL-36RA) [5]. IL-36RA inhibits IL-36 receptor (IL-36R) activation by the pro-inflammatory IL-36α, -β, and -γ. Consistently, an IL-36R-blocking antibody is now approved for GPP [6, 7]. Keratinocytes are the main IL-36R-expressing cells and the major source of IL-36γ in psoriatic lesions [7]. IL-36γ is produced as an inactive precursor activated by Cathepsin S and recently linked to gasdermin-mediated secretion [8, 9].

IL-1β, the prototypical IL-1 family cytokine, activates the ubiquitously expressed IL-1 receptor type I (IL-1R1), that is also activated by IL-1α, another IL-1 family member [10]. Dysregulated IL-1 signaling, for instance due to mutations in *IL1RN* (encoding the IL-1 receptor antagonist (IL-1RA)), contributes to pustular skin eruptions resembling psoriasis [11–13]. Although genetic and experimental evidences link IL-1 signaling to psoriasis, the precise cellular and molecular mechanisms underlying the function of this pathway remain unclear [14–16].

IL-1β is activated by caspase-1 upon inflammasomes assembly [17]. Inflammasomes are multiprotein complexes comprising a sensor, such as NLRP1 (NOD-like receptor family pyrin domain-containing protein 1), NLRP3 or AIM2 (absent in melanoma 2), the adaptor protein ASC (apoptosis-associated speck-like protein containing a CARD), and caspase-1. Upon activation induced by specific stressors, sensors oligomerize and recruit ASC, which forms aggregates (ASC specks), inducing caspase-1 self-activation [17]. Caspase-1 then cleaves and activates proIL-1β and proIL-18, and gasdermin D (GSDMD), which forms pores inducing cytokine release and pyroptosis, a lytic form of cell death [18]. Like IL-36γ, IL-1β and IL-18 lack signal peptides and rely on this non-canonial secretion pathway. While essential for host defense, inflammasomes also contribute to common inflammatory diseases [19].

NLRP1 is the main inflammasome sensor in human keratinocytes [20] and SNPs in *NLRP1* are associated with skin-inflammatory diseases including psoriasis [21]. NLRP1 is activated downstream of the ribotoxic stress response (RSR) by UVB, toxins, and certain antibiotics [22–25] or by viral infection through 3 C protease cleavage or by binding of long double-stranded RNA (dsRNA) [26, 27]. In contrast, binding of oxidized thioredoxin or dipeptidyl peptidase (DPP) 8/9 inhibits NLRP1, whereas DPP8/9 inhibitors activate it [28, 29]. Importantly, the NLRP1 pathway is poorly conserved in murine keratinocytes [30, 31], limiting studies in mice.

Despite the genetic link of NLRP1 to psoriasis and the roles of IL-1β and IL-36γ in the disease, a possible function of NLRP1 expressed by keratinocytes in the pathophysiology of psoriasis remains unexplored.

To address this, we pharmacologically activated NLRP1 in human primary keratinocytes (HPKs) cultured in human skin equivalents (SEs) [32]. NLRP1 activation in SEs induced an IL-1-dependent phenotype with key features of psoriasis. We identified IL-36γ as an NLRP1-induced cytokine released via GSDMD pores and demonstrated active NLRP1 signaling in psoriatic keratinocytes.

Known triggers of NLRP1, such as UVB, toxins, or viral infection, are unlikely to be relevant in psoriasis. Instead, endogenous dsRNA is increasily recognized as a stress factor in several inflammatory conditions [33]. Normally restricted to the nucleus and mitochondria, dsRNA is released into the cytoplasm and extracellular space upon stress, acting as damage- or homeostasis-associated molecular pattern (DAMP/HAMP) [34]. In the skin, dsRNA contributes to inflammation and repair via TLR3 [35, 36]. Small nuclear U1 spliceosomal RNA (U1 RNA), a short 164-base enodogenous RNA component of nuclear ribonucleoprotein complexes, is released by keratinocytes after injury and suggested to be involved in psoriasis [37]. Cytoplasmic dsRNA is also elevated in psoriasis due to reduced adenosine-to-inosine (A-to-I) RNA editing [38]. We discovered that short enougenous dsRNA, such as U1 RNA, can activate NLRP1 under inflammatory conditions, suggesting a physiologically relevant trigger in psoriatic keratinocytes.

Collectively, our findings reveal a central role for NLRP1 inflammasome activation in keratinocyte-driven inflammation in psoriasis and highlight NLRP1-activated SEs as a relevant model for studying disease mechanisms and potential therapies.

## Results

### SEs with CRISPR/Cas9-modified HPKs represent a physiological in vitro skin model

**Fig. 1:**
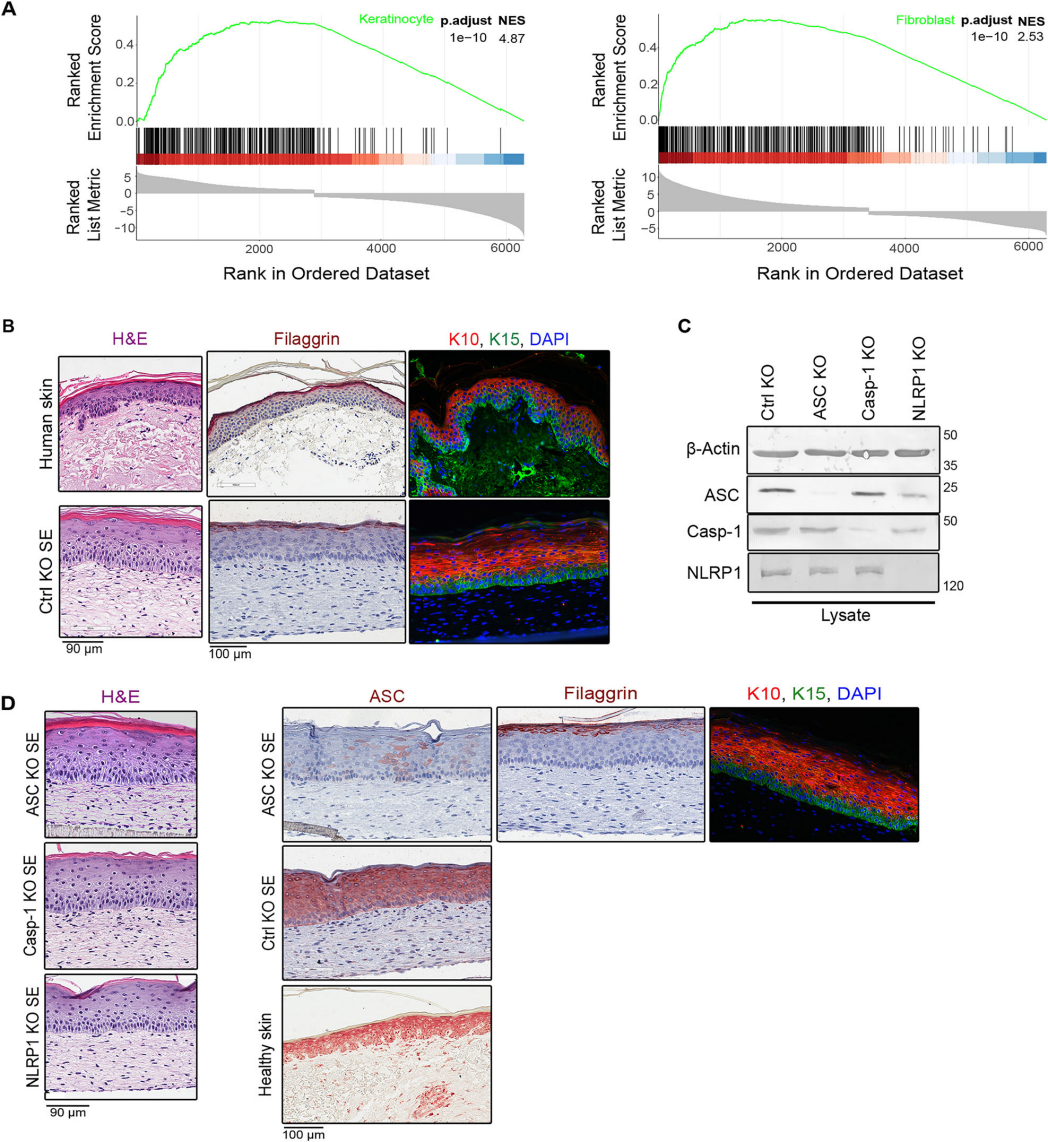
Skin equivalents (SEs) with CRISPR/Cas9-modified HPKs resemble human skin. SEs were generated with CRISPR/Cas9-modified polyclonal HPKs. A control non-targeting sgRNA was used (Ctrl KO) or sgRNAs targeting ASC, caspase-1, or NLRP1. **A** GSEA shows that gene expression profiles of control HPKs (left) or HDFs (right) within SEs closely resemble those of the epidermis and dermis in human skin. Genes were ranked by differential expression in HPKs over HDFs for the epidermis and vice versa for the dermis of the SEs. Gene sets “keratinocytes” and “fibroblasts” were obtained from [41]. Red regions indicate positive NES and enrichment of the corresponding gene set. (**B**, **C**, **D)** Control and NLRP1 inflammasome-deficient HPKs form a stratified epidermis in SEs. **B** H&E, immunohistochemical or immunofluorescence analysis of SEs with Ctrl KO HPKs and healthy human skin, showing the expression of filaggrin and keratin10 and -15 (K10, K15). Nuclei are counterstained with DAPI. Scale bars, 90 µm or 100 µm. **C** Western blot confirming loss of the indicated proteins in polyclonal knockout HPKs. Expression of β-actin served as control. **D** Left: H&E staining of SEs generated with ASC, caspase-1 or NLRP1 knockout HPKs. Scale bar, 90 µm. Right: Immunohistochemical or immunofluorescence staining of SEs generated with ASC knockout HPKs for expression of ASC, filaggrin, K10 and K15. SEs generated with Ctrl KO HPKs and healthy human skin are used as controls for ASC staining. Nuclei are counterstained with DAPI. Scale bar, 100 µm. Data in **B**, **C**, **D** are representative of 3 independent experiments. GSEA, gene set enrichment analysis; H&E, hematoxylin and eosin; HDFs, human dermal fibroblasts; HPKs, human primary keratinocytes; KO, knockout; NES, normalized enrichment score; SE, skin equivalent.

Because murine keratinocytes do not express significant levels of NLRP1 or IL-1β [30, 31],we used organotypic skin equivalents (SEs) generated with human primary fibroblasts (HPFs) and keratinocytes (HPKs) as a physiological model for human skin [39]. A recently developed full-thickness, fibroblast-derived matrix SE recapitulates human epidermal stratification and differentiation [32, 40]. We generated SEs using polyclonal CRISPR/Cas9 control-knockout HPKs, separated epidermis and dermis enzymatically, and performed RNA sequencing. Gene-expression profiles closely matched those of keratinocytes and fibroblasts of human skin (Fig. 1A) [41] and histological analysis confirmed proper epidermal differentiation (Fig. 1B).

We then generated polyclonal CRISPR/Cas9 HPKs lacking expression of NLRP1, ASC, or caspase-1 and found that all knockout HPKs formed a stratified epidermis indistinguishable from controls (Fig. 1C, D).

### Talabostat treatment induces NLRP1 activation in suprabasal keratinocytes

**Fig. 2:**
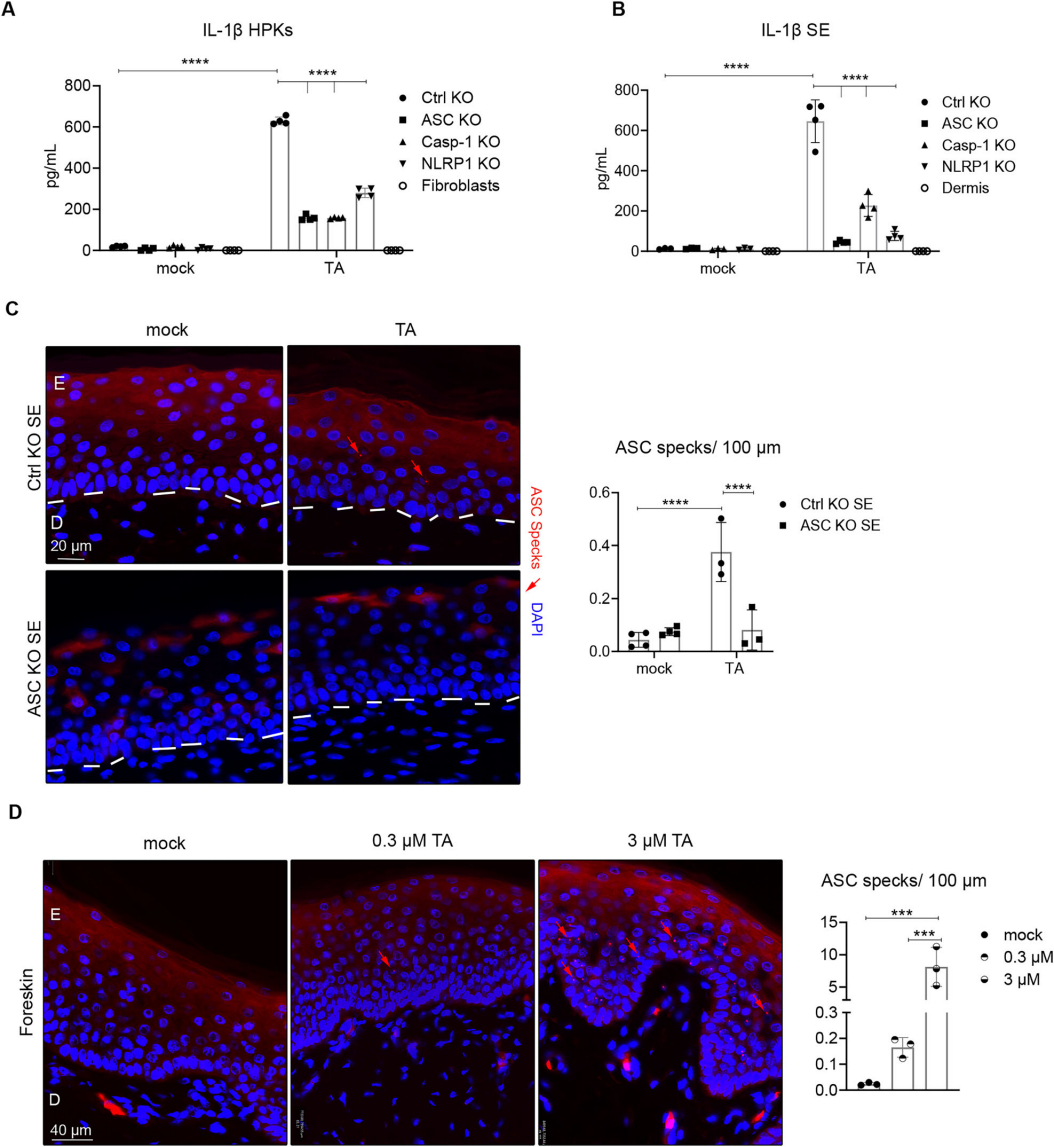
Talabostat activates the NLRP1 inflammasome in suprabasal keratinocytes. Control or knockout HPKs were treated with talabostat for 3 days in (**A**) monoculture (3 µM) or (**B**) SEs (0.3 µM) and analyzed for secretion of IL-1β by ELISA. **A** HDFs or (**B**) dermal equivalents (dermis) served as controls. **C** Immunofluorescence staining for ASC in SEs generated with Ctrl or ASC KO HPKs 3 days after mock- or treatment with talabostat (0.3 µM). ASC specks (red arrows) were quantified. Scale bar, 20 µm. **D** ASC immunofluorescence staining of healthy human skin mock-treated for 3 days ex vivo or with the indicated concentrations of talabostat. ASC specks were quantified. Scale bar, 40 µm. Data are represented as mean ± SD of (**A**) 4 or (**B**, **C**, **D**) ≥ 3 independent experiments. P values were calculated with (**A**, **B**, **C**) two-way ANOVA and (**D**) one-way ANOVA. (∗∗∗∗*P* < 0.0001, ∗∗∗*P* ≤ 0.001, ∗∗*P* ≤ 0.01, and ∗*P* ≤ 0.05, ns = not significant). TA, talabostat; HPKs, human primary keratinocytes; HDFs, human dermal fibroblasts; SE, skin equivalent.

NLRP1 is constitutively inhibited in keratinocytes by DPP8/9 binding [42]. Because talabostat selectively inhibits DPP8/9 and directly activates NLRP1 [28], we used it as a pharmacological stimulus to activate NLRP1 inflammasome in HPKs [20]. In 2D cultures talabostat induced caspase-1 activation and robust secretion of mature IL-1β, which depended on NLRP1, ASC, and caspase-1 expression (Fig. 2A and Fig. S1A). Similarly, talabostat triggered mature IL-1β secretion from HPKs in SEs, but not from dermal equivalents, and this response was strongly reduced in SEs with NLRP1-, ASC-, or caspase-1-deficient HPKs (Fig. 2B and Fig. S1B). We next assessed ASC speck formation as a second readout of inflammasome activation [17]. Unexpectedly, ASC specks were detected exclusively in suprabasal keratinocytes within talabostat-treated SEs (Fig. 2C). Although NLRP1 can be efficiently activated in proliferating HPKs in 2D monoculture (Fig. S1C) [24, 28], talabostat induced ASC specks in differentiated suprabasal keratinocytes also in human skin ex vivo (Fig. 2D), indicating that the SEs are a good model for NLRP1 activation in human epidermis.

Together, these results show that talabostat is a specific activator for NLRP1 in SEs and that this 3D system represents a physiologically relevant model to address the roles of the NLRP1 inflammasome in human skin.

### NLRP1 activation induces a strong pro-inflammatory signature, predominantly in dermal fibroblasts

**Fig. 3:**
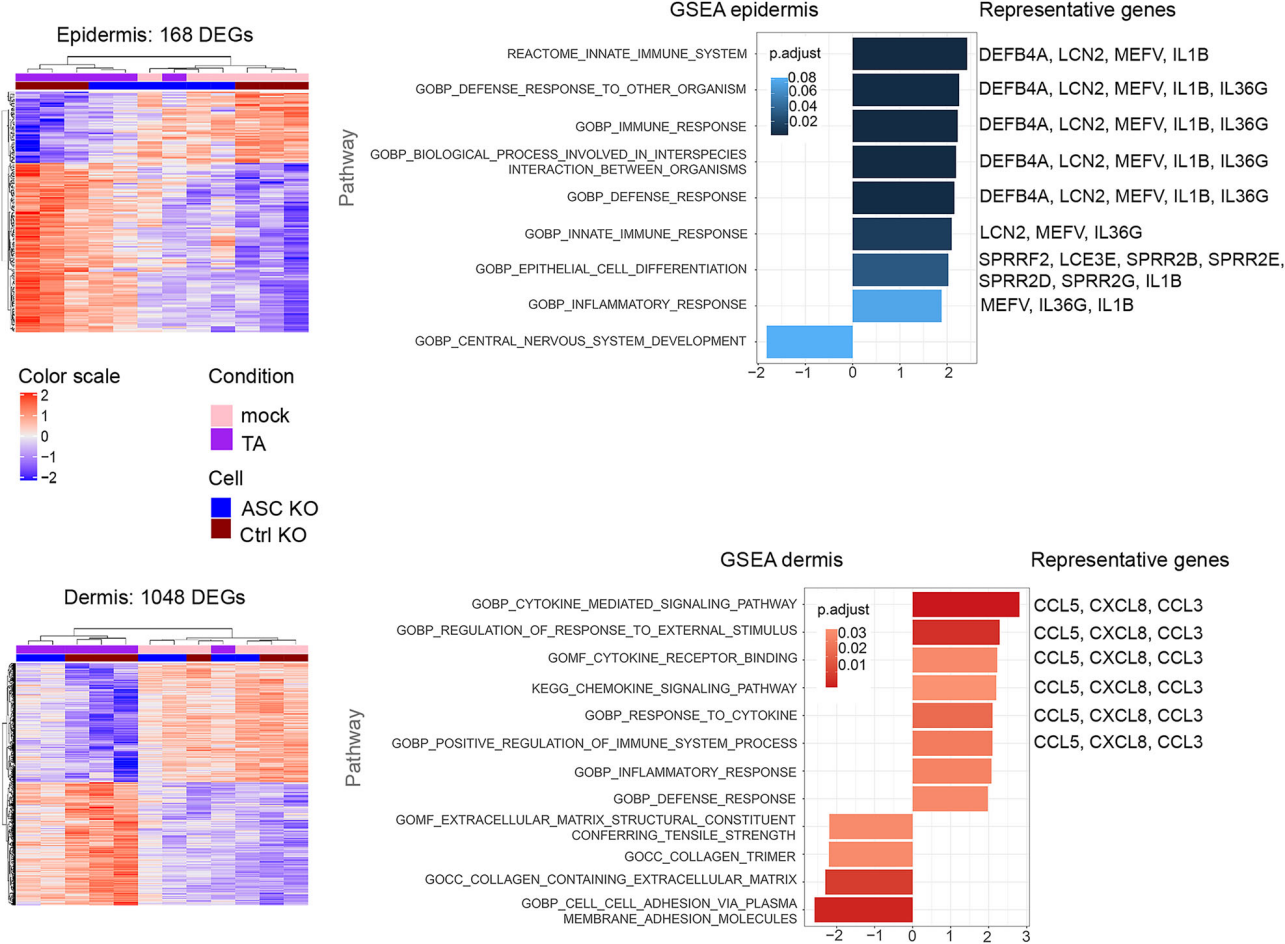
NLRP1 activation in SEs induces a pro-inflammatory signature. SEs with control or ASC knockout HPKs (triplicates) were mock- or talabostat-treated (0.3 μM) for 3 days. Epidermis and dermis were separated and mRNA expression characterized by RNA-seq. Left: heatmaps of keratinocytes with 168 differentially expressed genes and of fibroblasts with 1048 genes, upon talabostat-induced ASC-dependent NLRP1 activation. Right: GSEA reveals strongly induced and repressed pathways. Representative genes are leading edge genes of these pathways chosen for further analysis. SE, skin equivalent; HPKs, human primary keratinocytes, DEGs: differentially expressed genes; GSEA: gene set enrichment analysis.

To investigate the transcriptional consequences of NLRP1 activation, SEs with control or ASC-deficient HPKs were mock-treated or with talabostat for 3 days, followed by epidermal-dermal separation and RNA-seq. ASC-deficient HPKs were used because ASC knockout most efficiently abolished IL-1β secretion (Fig. 2B, Fig. S1B). Dispase II digestion allowed efficient separation of epidermis and dermis (Fig. S2A). Upon RNA-seq analysis, only genes regulated by talabostat in control cells and, therefore, by NLRP1 inflammasome activation, were considered (see Supplementary Methods). Talabostat significantly regulated 168 genes in epidermal keratinocytes in an ASC-dependent manner (Fig. 3), including differentiation markers (*TGM1, SPRRs*) and innate immune effectors (*IL1B*, *IL36G*, defensins, S100 proteins*)* (Fig. 3, Fig. S3).

Surprisingly, 1048 genes were significantly induced or suppressed in dermal fibroblasts, mainly involved in immune defense and cytokine-responsive pathways (*CCL*, *CXCL* chemokines, MMPs, *IL36A*, *IL36B*, *IL8*) (Fig. 3, Fig. S3). Because NLRP1 activation is restricted to keratinocytes, the dermal response is likely driven by paracrine IL-1β/IL-18 signaling.

These results demonstrate that NLRP1 activation in keratinocytes is sufficient to induce a robust pro-inflammatory signature in SEs lacking immune cells, suggesting a mechanism for local inflammation and immune cell recruitment in human skin.

### IL-1 is the main effector of NLRP1 inflammasome activation and induces IL-36γ expression

To validate our RNA-seq findings obtained from a single donor, we generated SEs with HPKs from 5 additional donors and analyzed mRNA levels of representative genes after talabostat treatment. Despite inter-donor variability, all tested genes showed consistent upregulation (Fig. S4A), confirming that NLRP1 activation induces a robust and donor-independent transcriptional response. Talabostat-induced gene regulation was abolished not only by ASC knockout but also by NLRP1 knockout (Fig. S4B).

**Fig. 4:**
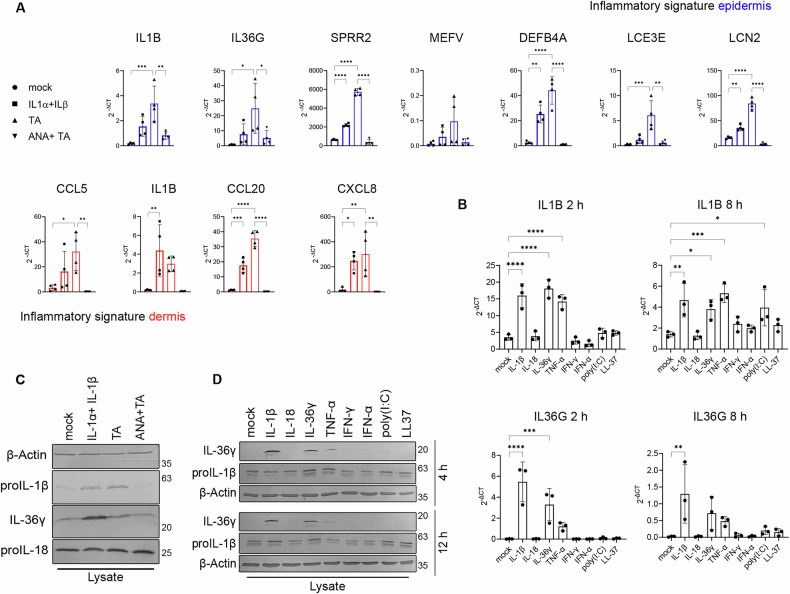
IL-1 is major effector of NLRP1 inflammasome activation is SEs. **A**, **C** SEs were mock-treated or with IL-1α/β (1 ng/ml each), talabostat (0.3 μM), or anakinra (10 μg/ml) plus talabostat, for 3 days. **A** mRNA levels of representative genes of the epidermis (blue) and of the dermis (red) were determined by qPCR related to HPRT expression. **C** Protein expression of IL-1β, IL-36γ and IL-18 was analyzed in the epidermis of SEs 3 days after treatment by western blot. Expression of β-actin served as control. **B**, **D** Starved HPKs from 3 different donors in monolayer were treated with IL-1β (10 ng/ml), IL-18 (20 ng/ml), IL-36γ (100 ng/ml), TNFα (10 ng/ml), IFN-γ (20 ng/ml), IFN-α (10 ng/ml), poly(I:C) (1 μg/ml), or LL-37 (1 μg/ml). IL-1β and IL-36γ expression was determined at the (**B**) RNA level 2 h (upper panel) and 8 h (lower panel) after stimulation and (**D**) at the protein level after 4 h and 12 h. HPRT and β-actin expression were used as internal controls, respectively. **A** Data are represented by mean ± SD of 4 replicates and are representative of 2 independent experiments, or (**B**) 3 different donors, or a representative blot out of (**C**) 2 or (**D**) 3 donors is shown. *P* values were calculated with one-way ANOVA (∗∗∗∗*P* < 0.0001, ∗∗∗*P* ≤ 0.001, ∗∗*P* ≤ 0.01, and ∗*P* ≤ 0.05, ns = not significant). ANA, anakinra; TA, talabostat. HPKs, human primary keratinocytes ; SE, skin equivalent.

To identify the main driver of this inflammatory signature, we examined IL-1α/β, both activating IL-1RI. SEs were treated with recombinant IL-1 (at levels matching talabostat-induced IL-1α/β), talabostat, or talabostat plus anakinra. IL-1 induced gene expression more rapidly than talabostat at day 1, particularly in the epidermis (Fig. S5A), whereas talabostat caused stronger upregulation by day 3 (Fig. 4A). Anakinra inhibited talabostat-induced expression at both time points (Fig. 4A, Fig. S5A), demonstrating that IL-1 is the central effector driving the NLRP1-dependent transcriptional regulation. Among IL-1-regulated genes, we focused on IL-36γ. In 2D-cultured HPKs, IL-1β and IL-36γ strongly induced *IL36G* mRNA and protein, whereas *IL36G* was not expressed under basal conditions, demonstrating a crucial role of IL-1β in regulating IL-36γ expression (Fig. 4B, D). Inflammasome components, including *IL1B*, were constitutively expressed and further upregulated by IL-1β or IL-36γ (Fig. 4B, D and Fig. S5B).

In the epidermis of SEs, IL-1 stimulation resulted in the highest IL-36γ protein levels despite modest mRNA induction, whereas talabostat caused strong mRNA upregulation but only intermediate protein levels (Fig. 4A, C), suggesting a regulation of IL-36γ upon NLRP1 activation beyond transcriptional induction.

### NLRP1 inflammasome activation regulates IL-36γ secretion through GSDMD pores

**Fig. 5:**
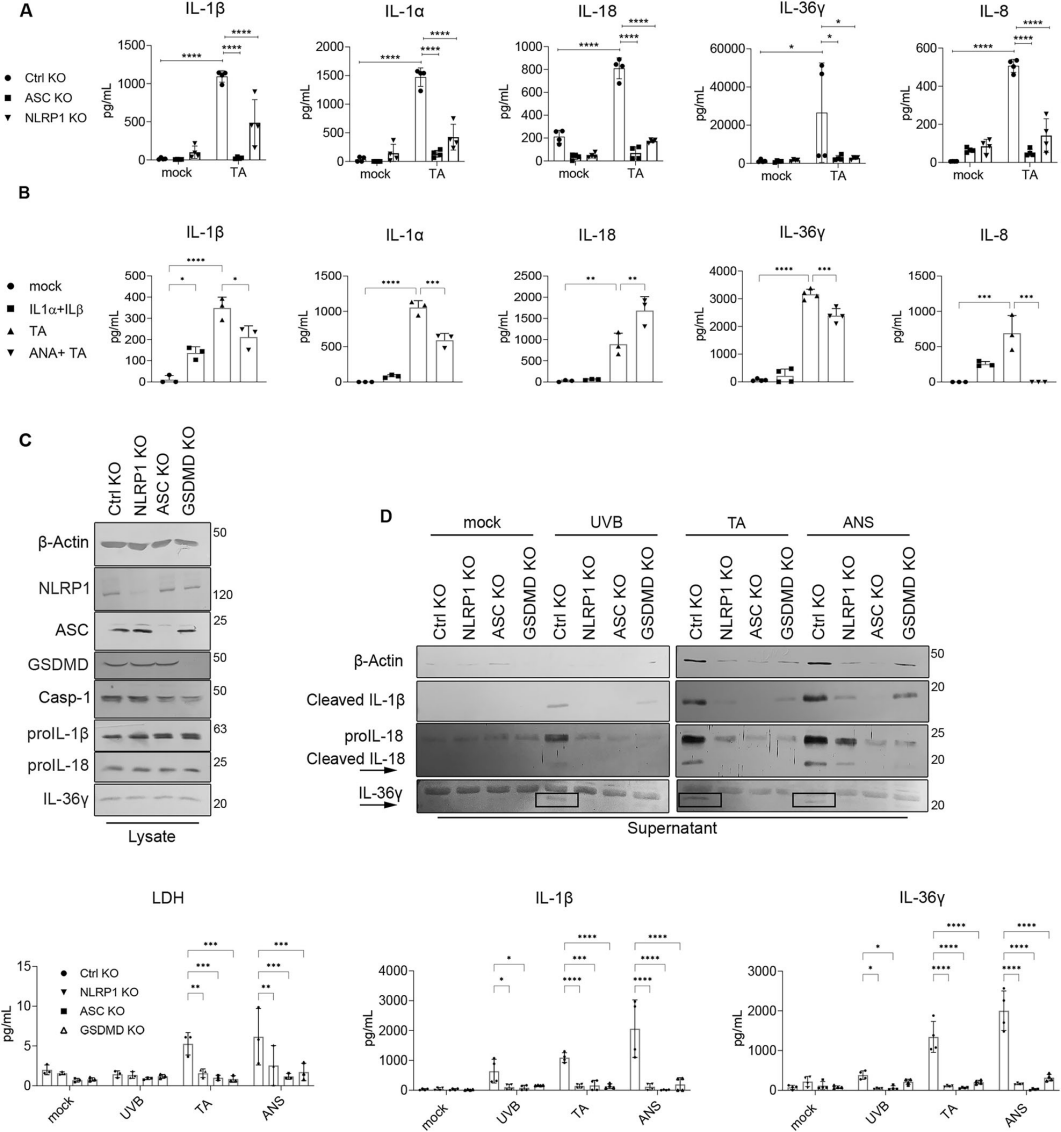
NLRP1 activation regulates IL-36γ release. SEs with (**A**) control, ASC, or NLRP1 knockout HPKs or (**B**) wild type HPKs were treated for 3 days. Treatments were: (**A**) mock or talabostat (0.3 µM), (**B**) mock, IL-1α/β (1 ng/ml each), talabostat (0.3 μM), or anakinra (10 μg/ml) plus talabostat. Cytokine release was measured by ELISA. **C** Western blot showing protein expression in mock-treated polyclonal knockout HPKs in 2D monoculture, primed overnight with IL-1α (10 ng/ml) for the induction of IL-36γ expression. **D** Knockout HPKs from 3 donors in 2D monolayer were primed with IL-1α, then either mock-treated, UVB irradiated (86.4 mJ/cm^2^, 6 h), treated with talabostat (3 μM, 12 h), or with anisomycin (1 μM, 5 h). Protein release was analysed by western blot (upper panel) or ELISA (lower panel). Expression of β-actin in the mock lysate served as loading control for (**C**) and (**D**). **D** Pyroptosis was assessed by LDH release. **A**, **B** Data are represented by mean ± SD of ≥3 replicates and are representative of 2 experiments. **C**, **D** Data are represented by mean ± SD of ≥ 3 donors (LDH 3 donors, ELISA 4 donors) or a representative blot out of 3 is shown. **A**, **D** *P* values were calculated with two-way ANOVA and (**B**) with one-way ANOVA (∗∗∗∗*P* < 0.0001, ∗∗∗*P* ≤ 0.001, ∗∗*P* ≤ 0.01, and ∗*P* ≤ 0.05, ns = not significant). SE, skin equivalent; HPKs, human primary keratinocytes; TA: talabostat,; ANA: anakinra.

To dissect the regulation of IL-36γ in NLRP1-activated SEs, we measured IL-36γ, IL-1α, IL-1β, IL-18, and IL-8 in supernatants of mock- or talabostat-treated SEs (Fig. 5A). Cytokine release was strongly induced upon NLRP1 activation in control SEs and significantly reduced in ASC- or NLRP1-deficient SEs (Fig. 5A). We then analyzed whether the release of these cytokine was IL-1 dependent. We found that IL-8 secretion was entirely IL-1 dependent, as shown by induction upon recombinant IL-1 treatement and suppression by anakinra (Fig. 5B), in line with its IL-1-driven transcription (Fig. 4A) and known roles in keratinocytes and fibroblasts [43, 44]. However, although IL-36γ mRNA and intracellular protein were strongly upregulated by IL-1 or talabostat in HPKs within SEs (Fig. 4A, C), secretion occurred only upon NLRP1 activation and was significantly reduced by anakinra (Fig. 5B), indicating that release requires inflammasome activation.

To directly test this, HPKs from three donors were primed with IL-1α to induce stable IL-36γ expression before NLRP1 activation with UVB, talabostat or anisomycin (Fig. 5C, D). IL-36γ secretion was strictly dependent on NLRP1, ASC and GSDMD, demonstrating that IL-36γ, which lacks a signal peptide, is released through GSDMD pores. Cathepsin S, the main IL-36γ-activating protease, was constitutively expressed and further induced by IFN-γ and, to a lesser extent, IL-1β (Fig. S6A, B). HPKs primed with IL-1α (to induce IL-36γ) and IFN-γ (to induce cathepsin S), released processed cathepsin S only upon NLRP1 activation, and not the full-length form, in an NLRP1-, ASC- and GSDMD-dependent manner (Fig. S6C, D), indicating co-release with IL-36γ through GSDMD pores.

Together, these results demonstrate that NLRP1 inflammasome activation regulates IL-36γ at two levels: (i) IL-1-dependent induction (and, to a lesser extent, cathepsin S) and (ii) GSDMD-dependent release of both IL-36γ and its activating protease.

### NLRP1 activation in SEs induces a psoriasis-like molecular and histological phenotype in the epidermis

**Fig. 6:**
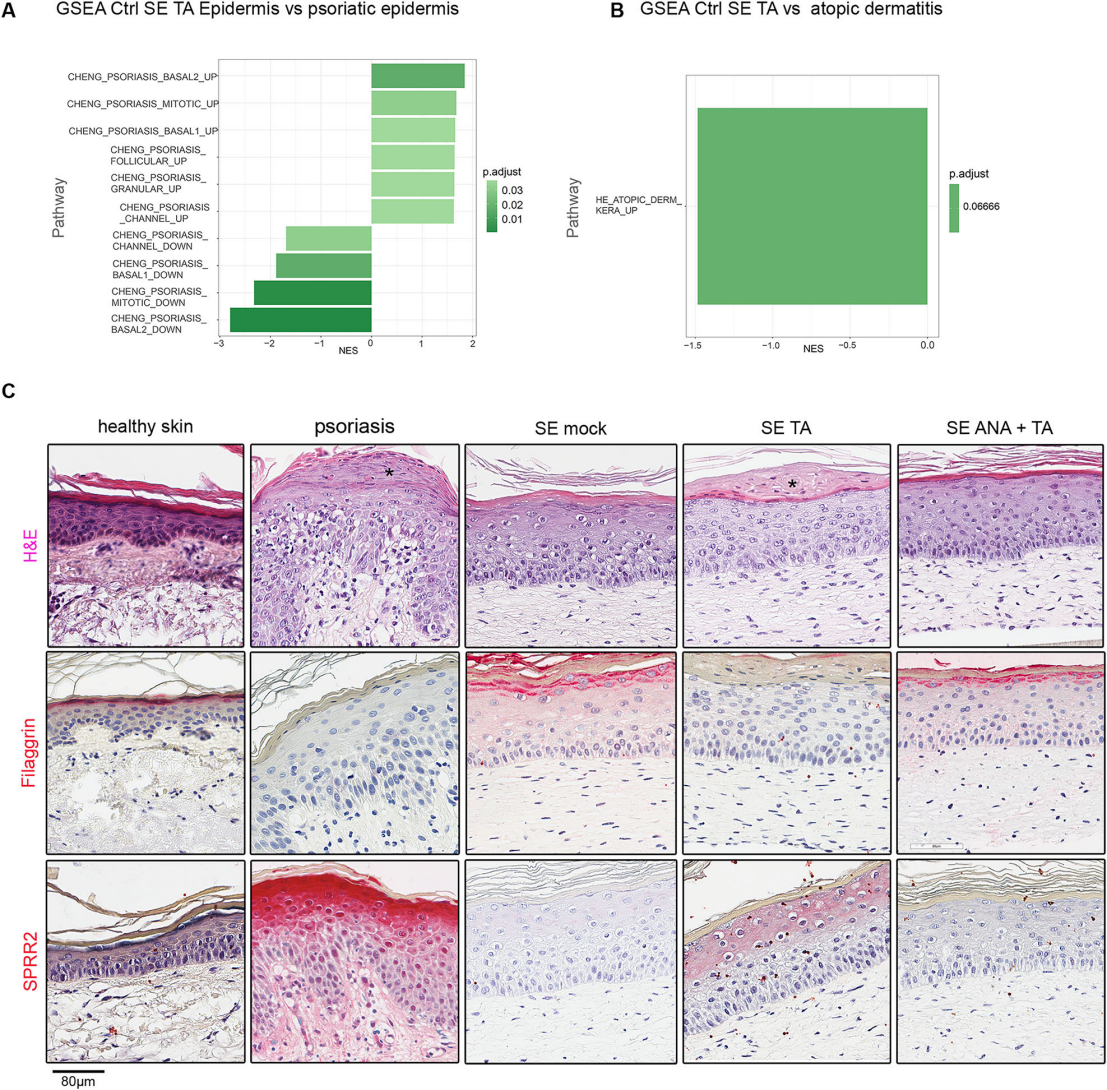
NLRP1 activation in SEs induces a phenotype resembling psoriasis. Talabostat-treated SEs with control HPKs were compared to psoriatic lesions at both mRNA and histological levels. GSEA was performed on mRNA from control SEs treated with talabostat (0.3 µM, 3 days) versus (**A**) psoriatic epidermis [45], or (**B**) atopic dermatitis [46]. Gene sets significantly upregulated in psoriatic keratinocytes (positive NES) were also upregulated in the epidermis of talabostat-treated SEs, whereas no significant enrichment was observed for atopic dermatitis. **C** Histological comparison of healthy and psoriatic skin, and SEs treated with mock, talabostat, or anakinra plus talabostat. Staining includes H&E, filaggrin and SPRR2; parakeratosis is indicated (*). Scale bar, 80 µm. Stainings are representative of ≥3 independent experiments (SEs) and 3 donors of healthy and psoriatic skin. SE, skin equivalent; HPKs, human primary keratinocytes; GSEA, gene set enrichment analysis; TA, talabostat.

Because expression of IL-36γ, linked with psoriasis [7], is strongly induced upon NLRP1 activation in SEs, we compared gene expression in SE keratinocytes after talabostat treatment with keratinocytes from lesional psoriasis or atopic dermatitis (as control) (Figs. 6A, B) [45, 46]. GSEA revealed shared regulation of genes and pathways between the epidermis of talabostat-treated SEs and psoriatic keratinocytes, but not atopic dermatitis (Fig. 6A, B and Fig. S7). Histological and immunohistochemical analysis further confirmed that the epidermis of talabostat-treated SEs recapitulates features of psoriatic epidermis (Fig. 6C). Parakeratosis, characterized by retention of nuclei in the *stratum corneum* due to accelerated keratinocyte proliferation and differentiation, was induced by talabostat but absent in mock-treated SEs. Filaggrin expression in the granular layer was markedly reduced, while SPRR2, a keratinocyte differentiation marker, was upregulated, mirroring psoriatic lesions. Notably, this phenotype was IL-1-dependent, as anakinra pretreatment prevented parakeratosis and associated molecular changes (Fig. 6C). Talabostat-treated SEs did not show keratinocyte hyperproliferation and acanthosis, a typical thickening of the *stratum spinosum* observed in psoriatic lesions, likely due to the use of serum-reduced medium, as reported in other psoriasis models based on SEs [47].

Overall, these results demonstrate that NLRP1 activation and IL-1 signaling in SEs induce a molecular and histological signature closely resembling psoriatic epidermis.

### Inflammasome activation in psoriatic lesions

The IL-1 family members IL-1β and IL-36γ play important roles in psoriasis pathogenesis [7, 16]. Here, we show that IL-36γ, which lacks a signal peptide, is released via NLRP1 inflammasome activation in HPKs (Fig. 5D). NLRP1 activation in SEs also induced the release of several inflammatory mediatiors including IL-17 and IL-23, two key cytokines in psoriasis (Fig. S8). Since keratinocytes are the main source of IL-36γ in psoriasis, we hypothesized that inflammasome activation occurs in keratinocytes in vivo.

**Fig. 7:**
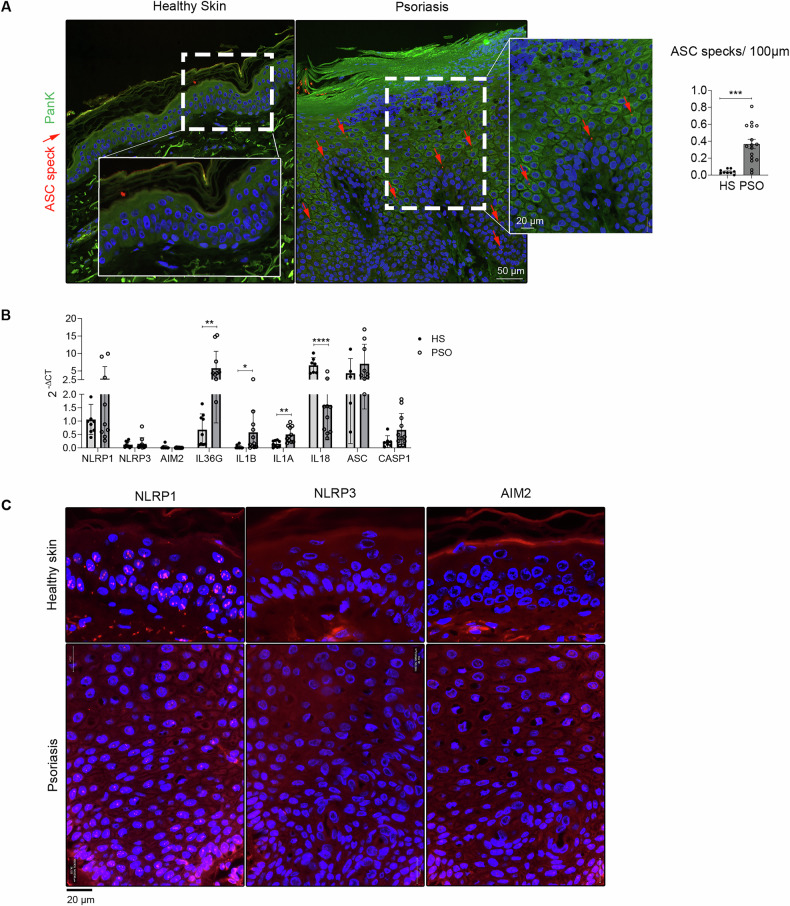
Inflammasome activation and NLRP1 expression in lesional psoriasis. **A** Tissue sections of healthy skin or lesional psoriasis were stained for ASC (red) and pan-keratin (green). Nuclei were counterstained with DAPI (blue). Merge pictures are shown. ASC specks are indicated by arrows and were quantified. Scale bar, 50 or 20 µm. Stainings are representative of 9 donors of healthy skin and 16 donors of psoriasis. **B** RNA was extracted from FFPE blocks of different donors of healthy skin or lesional psoriasis and expression of the indicated genes was quantified by RT-qPCR, related to HPRT expression. **C** RNAscope for NLRP1, NLRP3 and AIM2 (red dots) was performed with biopsies of healthy donors or those suffering from psoriasis. Nuclei were counterstained with DAPI. Representative merged pictures are shown. Scale bar, 20 µm. **A**, **B** P values were calculated with two-tailed unpaired *t*-test (∗∗∗∗*P* < 0.0001, ∗∗∗*P* ≤ 0.001, ∗∗*P* ≤ 0.01, and ∗*P* ≤ 0.05, ns = not significant). FFPE, formalin-fixed paraffin-embdeed; HS, healthy skin; PSO, psoriatic lesion.

All IL-1-generating human inflammasomes form ASC oligomers and recruit and activate caspase-1 [17]. We therefore examined ASC speck formation in tissue sections of healthy skin and psoriatic lesions. ASC-positive keratinocytes were abundant in psoriatic lesions but rare in healthy skin (Fig. 7A), indicating active inflammasome signaling. Although NLRP1 is the main inflammasome sensor in human keratinocyte [20], NLRP3 or AIM2 may also contribute to inflammation in psoriasis [16, 48–50]. mRNA analysis revealed high NLRP1 but minimal NLRP3 or AIM2 expression, together with significantly increased IL1B, IL1A, and IL36G levels in psoriasis compared to healthy skin (Fig. 7B). RNAscope further confirmed NLRP1 expression in keratinocytes, whereas NLRP3 and AIM2 were undetectable (Fig. 7C).

These data indicate that inflammasome activation, most likely driven by NLRP1, the only sensor detected, occurs in suprabasal keratinocytes of psoriatic lesions and may contribute to disease pathogenesis.

### Endogenous dsRNA activates NLRP1 in HPKs

**Fig. 8:**
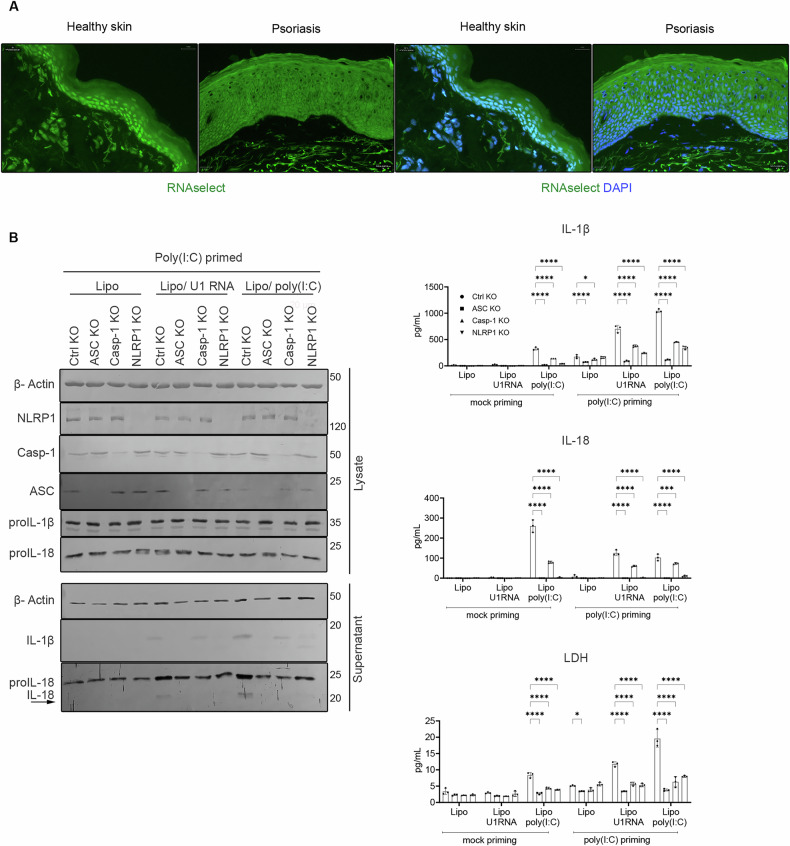
U1 RNA activates the NLRP1 inflammasome. **A** Tissue sections of healthy skin or lesional psoriasis were stained for RNA with SYTO RNASelect (green). Nuclei were counterstained with DAPI (blue). Scale bar, 20 µm. Stainings are representative of 3 donors of healthy skin and 3 of psoriasis. **B** Western blot for expression of the indicated proteins by polyclonal knockout HPKs from 3 different donors in 2D monolayer. Representative blot out of 3 is shown. HPKs were primed overnight with poly(I:C) (1 μg/ml) or mock-treated and then transfected with Lipofectamine (Lipo), Lipo/U1 RNA (1 μg/ml) or Lipo/poly(I:C) (1 μg/ml), for 8 h. Protein expression and release was determined by western blot (left panel) or ELISA (right panel). Expression of β-actin in the lysate was used as loading control. Cell death was measured by LDH release (right panel). **B** Data are represented by mean ± SD of 3 independent donors and are representative of 3 independent experiments. P values were calculated by two-way ANOVA (∗∗∗∗*P* < 0.0001, ∗∗∗*P* ≤ 0.001, ∗∗*P* ≤ 0.01, and ∗*P* ≤ 0.05, ns = not significant).

To investigate potential disease-relevant triggers of NLRP1 in keratinocytes, we focused on endogenous dsRNA, a stress factor involved in inflammatory diseases including psoriasis [33, 37, 38]. Consistent with previous observations [37], we detected accumulation of cytoplasmic RNA in psoriatic keratinocytes, in contrast to its predominantly nuclear localization in healthy skin (Fig. 8A). Among endogenous dsRNA, U1 RNA, a short 164 base nuclear RNA, has been associated with psoriasis [37]. To test whether short endogenous dsRNAs can activate NLRP1, HPKs were primed with the TLR3 agonist poly(I:C) prior to transfection of U1 RNA. Under these inflammatory conditions, U1 RNA induced NLRP1 activation, as shown by IL-1β, IL-18 and IL-36γ secretion and the induction of pyroptosis (Fig. 8B, Fig. S9). Cytokine release was dependent on GSDMD pores at 5 h post U1 RNA transfection, but not at 8 h (Fig. S9), suggesting potential compensatory mechanisms involving other gasdermins at later time points.

These findings indicate that U1 RNA, and potentially other short endogenous dsRNA, can activate NLRP1 in keratinocytes, suggesting that cytoplasmic endogenous dsRNA may serve as a relevant activator of NLRP1 in psoriatic lesions.

## Discussion

Our study identified the NLRP1 inflammasome in keratinocytes as a novel potential driver of psoriatic inflammation and establishes a mechanistic link between NLRP1 activation, IL-1 signaling, and IL-36γ regulation. Althogh *NLRP1* variants have been associated with inflammatory skin diseases, including psoriasis [21], and both IL-1β and IL-36γ are implicated in disease pathogenesis [6, 7, 11–13], the connection between NLRP1 activation, IL-1β production and IL-36γ regulation, and its functional consequences in human epidermis, have not been previoulsy elucidated. Using human skin equivalents with polyclonal CRISPR-edited keratinocytes and biopsies of psoriatic lesions, we demonstrate that NLRP1 activation is sufficient to induce a transcriptional and histological phenotype resembling psoriasis and that NLRP1 is the main inflammasome sensor expressed in human epidermis and most likely activated in psoriatic keratinocytes. Mechanistically, NLRP1 activation regulates IL-36γ at different levels: IL-1-dependent induction of transcription and GSDMD-dependent secretion of both IL-36γ and cleaved cathepsin S, its activating protease. We also demonstrate that NLRP1 in keratinocytes drives a robust inflammatory response in dermal fibroblasts, even in the absence of immune cells, characterized by induction of chemokines, cytokines and matrix-remodeling factors. This highlighs the capacity of keratinocytes to initiate an inflammatory environment and suggests that NLRP1 activation might cause the recruitment of immune cells in vivo. Consistently, talabostat-treated SEs showed transcriptional and histological hallmarks of psoriasis, including parakeratosis, altered differentiation, and IL-1/IL36 pathway activation. Thus, NLRP1-activated SEs represent a novel relevant human model to study keratinocyte and fibroblast-driven inflammation, particularly in psoriasis, and may facilitate the development of therapeutic NLRP1 inhibitors.

A key discovery of our work is that short endogenous dsRNA can activate NLRP1 under inflammatory conditions. Although NLRP1 is known to sense long dsRNA, either viral or synthetic (poly(I:C)) [27], an activation of NLRP1 by endogenous dsRNA has never been elucidate before. U1 RNA, a short nuclear dsRNA released into the cytoplasm under stress, is implicated in psoriasis [37]. We demonstrate that U1RNA can activate NLRP1 when keratinocytes are primed via TLR3, mimicking an inflammatory state. Because NLRP1 directly binds only long dsRNA [27], this suggests an indirect mechanism allowing short endogenous RNAs to trigger inflammasome activation, providing a molecular explanation for how RNA homeostasis disturbances drive IL-1 and IL-36-mediated inflammation. To our knowledge, this is the first demonstration that NLRP1 can sense cellular perturbation via short endogenous dsRNA. Finally, we detected ASC specks in suprabasal keratinocytes of psoriatic lesions providing direct evidence of inflammasome activation in vivo in keratinocytes in psoriasis. Together with high NLRP1 expression and minimal NLRP3 and AIM2 transcripts, these finding strongly implicate NLRP1 as the predominant inflammasome active in psoriatic epidermis, although roles of other inflammasome in keratinocytes cannot be excluded [50].

In summary, in this study we identified NLRP1 as a keratinocyte sensor of endogenous short dsRNA and a central coordinator of IL-1 and IL-36-mediated inflammation in psoriasis, positioning it as a promising therapeutic target with the potential for dual inhibition of IL-1 and IL-36. Given its activity in suprabasal keratinocytes, anatomically accessible, topical NLRP1 inhibition may offer a targeted and safe strategy to treat psoriatic skin inflammation.

## Materials and methods

### Human skin samples

Surplus skin biopsies from plastic-surgery patients and psoriatic patients (fresh or paraffin-embedded) were collected at the University Hospital Zürich after informed consent was provided. Among the samples, 10 were from psoriasis vulgaris cases and 6 from generalized pustular psoriasis. The study was approved by the Cantonal Ethics Committee of Zürich (KEK-ZH-Nr. 2015-0198; BASEC-Nr. 2024-01030) and conducted in accordance with the Declaration of Helsinki.

### Primary cell culture and treatments

Human primary keratinocytes (HPKs) were isolated as described [51], and cultured in keratinocyte serum-free medium with growth supplements. The HPKs used for skin equivalent generation were derived from foreskin biopsies obtained from pediatric donors (2-5 years of age). HDFs were isolated from abdominoplasty biopsies from adult female donors (30-40 years of age) as previously described [52], and cultured in DMEM with 10% FBS. HPKs were stimulated with talabostat, recombinant cytokines (IL-1β, IL-18, IL-36γ, TNF-α, IFN-γ), LL-37, poly(I:C), anisomycin, or UVB. DMSO or PBS served as controls. HDFs were used to produce dermal equivalents. Details are provided in the Supplementary Methods.

### CRISPR/Cas9-mediated gene knockout

HPKs were modified via lentiviral transduction [24], or electoporation of Cas9-sgRNA RNP complexes [53]. Knockout efficiency was validated by western blot. CRISPR-modified HPKs by lentivirus were used to generate skin equivalents. For detailed protocols and sgRNA sequences, see Supplementary Methods and Supplementary Table 1.

### Generation of skin equivalents

Skin equivalents were prepared as described [32]. Briefly, dermal equivalents were prepared by seeding HDFs onto transwell inserts and cultured in supplemented medium for 4 weeks. HPKs were seeded on top, cultured submerged for 3 days, then air-lifted for 2 weeks. SEs were treated 10 days post air-lift with talabostat, IL-1, anakinra, or control. For detailed protocol, see Supplementary Methods.

### Transfection of keratinocytes

HPKs in 2D were transfected with U1 RNA or poly(I:C) using Lipofectamin 2000 following the manufacturer’s instructions. For detailed protocol, see Supplementary Methods.

### Molecular analyses

#### Real-time PCR

Total RNA was isolated with Trizol from cells or SEs. cDNA was synhesized and analyzed by real-time PCR. HPRT served as reference gene. For detailed protocol and primer sequences see Supplementary Method and Supplementary Table 2.

#### Western blot

Monolayer cells, epidermis or dermis of SEs (after dispase II separation) were lysed in SDS-containing buffer. Supernatants were precipitated with acetone and pellets resuspendend in the same buffer. Proteins were separated by SDS PAGE and analysed with specific antibodies, as previously described [24]. For detailed protocol and antibodies specification, see Supplementary Methods and Supplementary Table 3.

#### ELISA and cytotoxicity

Cytokine release was quantified by ELISA and cytotoxicity was measured via LDH release assay, according to manufacturer’s instruction. For details see Supplementary Methods.

#### Cytokine array

SE supernatants were analysed using Human XL Cytokine Array. For detailed protocol see Supplementary Methods.

### Histology and immunostaining

Samples were fixed overnight in formalin 4%, paraffin-embedded and sectioned. H&E staining was performed for morphology. Immunohistochemistry and immunosluorescence were performed for protein localization. ASC specks were quantified using QuPath software. Epidermal thickness was measured with Aperio ImageScope. For detailed protocol and antibody specification see Supplementary Method and Supplementary Table 3.

### High-throughput RNA sequencing

RNA was isolated from the epidermis and dermis of SEs after enzymatic separation by dispase II. Libraries were prepared using Illumina TruSeq Standard mRNA kit. Sequencing was performed with Illumina Novaseq 6000 (25 M reads/sample). Gene-level counts were quantified from single-end reads aligned to the GRCh38.d1.vd1 genome using STAR v2.7.5a [54]. Differential expression was analysed with limma-vonn [55], and visualized with ComplexHeatmap [56]. Functional enrichment was analysed with clusterProfiler and GSEA [57]. For detailed protocol, see Supplementary Method.

### RNA-scope and RNA staining

RNAscope was performed using target-specific probes for NLRP1, AIM2, or NLRP3. Cytoplasmic RNA was visualized with SYTO RNASelect Green Fluorescent Cell Stain. Nuclei were counterstained with DAPI. For detailed protocol, see Supplementary Method.

### Statistical analysis

Statistical analysis for not high throughput data was performed using the software GraphPad Prism. Significance was set at *p* < 0.05. (∗∗∗∗*P* < 0.001, ∗∗∗*P* ≤ 0.001, ∗∗*P* ≤ 0.01, and ∗*P* ≤ 0.05, ns = not significant). Specific tests are indicated in figure legends.

## Supplementary information

Supplementary files_ all_ M&M_ Supplementary Figures & Legends

Supplementary Methods

## Data Availability

The gene expression data generated in this study were deposited in the Gene Expression Omnibus (GEO) under the accession number GSE282206.
