# Supplementary material for: NLRP1 inflammasome activation in skin equivalents reveals mechanistic insights into the roles of keratinocytes in psoriasis
Source: Cell Death Dis. 2026 May 30;17(1):670. doi: 10.1038/s41419-026-08908-6 (PMC13424314; doi:10.1038/s41419-026-08908-6)
Supplement: Supplementary file 1 — Supplementary files_ all_ M&M_ Supplementary Figures & Legends [file 41419_2026_8908_MOESM1_ESM.pdf]

Supplementary Figures

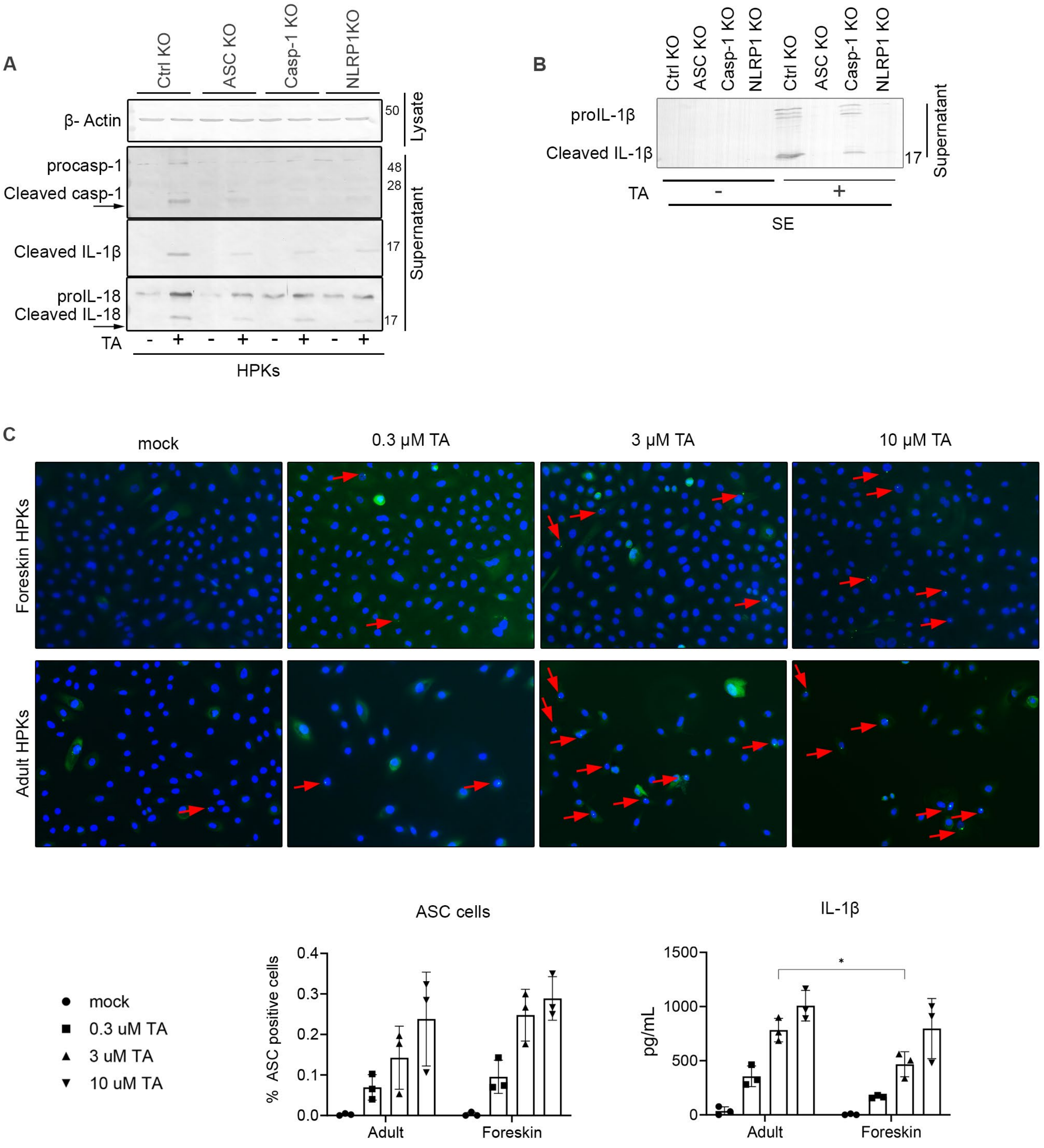

Fig S1A, B, C: Talabostat induces caspase-1 activation and secretion of mature IL-1β. (A)

Control or knockout HPKs were mock-treated or treated with talabostat (3 μM) for 3 days and

analyzed for caspase-1, IL-1β, and IL-18 activation and secretion by western blot. Expression of β-actin in the lysate was used as loading control. **(B)** SEs generated with control or knockout HPKs were mock-treated or treated with talabostat (0.3 μM) for 3 days, and IL-1β activation in the supernatant was assessed by western blot. **(C)** Foreskin and adult keratinocytes were mock-treated or with 0.3, 3 and 10 μM of TA for 1 day. ASC speck formation was assessed by immunofluorescence and quantified. The percentage of ASC-positive cells was determined by counting cells containing specks relative to the total number of cells across five independent microscopic fields, and values were averaged. Each dot represents an individual keratinocyte donor. IL-1β release was quantified by ELISA. ELISA data represent the average between 3 replicates from 3 different donors. P values were calculated with two-ways ANOVA. (∗∗∗∗P < 0.0001, ∗∗∗P ≤ 0.001, ∗∗P ≤ 0.01, and ∗P ≤ 0.05, ns = not significant).

Abbreviation: TA, talabostat; SE, skin equivalent; HPKs, human primary keratinocytes.

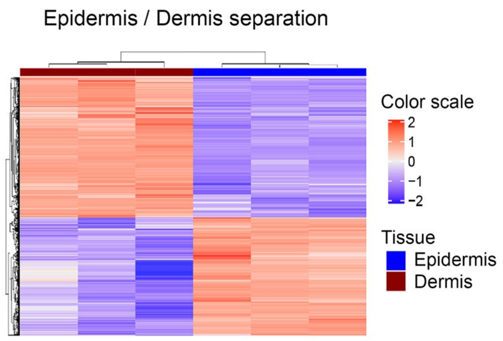

Fig S2: Heatmap showing separation of epidermis and dermis. Epidermis and dermis from SEs generated with control HPKs (triplicates) were separated with dispase II digestion and analyzed by RNA-seq. The heatmap of differentially expressed genes confirms efficient separation of the two skin layers and consistent gene expression profiles across replicates.

Abbreviation: SE, skin equivalent; HPKs, human primary keratinocytes.

Pathways Epidermis	Leading edge genes
REACTOME_INNATE IMMUNE_ SYSTEM	MUC4, PIGR, MUC16, DEFB4A, DEFB4B, ITGAX, SAA1, SERPINA1, LTF, ALOX5, MUC21, ITGB2, ATP6V0A4, CEACAM6, LCN2, MEFV, NCF2, RNASE7, CFB, IQGAP2, NFAM1, VNN1, NCF4, S100A12, CD177, CEACAM1, MUC20, RAC2, MUCL1, IL1B, ARG1, S100A7A, CTSS,LAT2, CD68, ICAM2,ENPP4, CRISPLD2, PI3
GOBP_DEFENSE_RESPONSE TO_OTHER_ORGANISM	SH2D1B, IL36A, DEFB4A, ITGAX, LCE3C, LTF, OASL, LCN2, MEFV, WFDC2, NCF2, RNASE7, IL23A, LCE3A, CFB, NRROS, VNN1, IL36G, A2M, GPER1, S100A12, CD177, KYNU, CEACAM1, PLA2G2F, SLA, IL1B, SERPINB4, ARG1, GSDMA, CX3CL1, IL36RN, NLRP10, GBP5, SERPINB9, IL36B, PI3, CXCL6, SRMS
GOBP_IMMUNE_ RESPONSE	SH2D1B, PIGR, IL36A, DEFB4A, IL1RL1, CSF3, VTCN1, LTF, CXCR4, ALOX5, ITGB2, OASL, LCN2, MEFV, WFDC2, IL19, NCF2, RNASE7, IL23A, CFB,NRROS, NFAM1, VNN1, IL36G, A2M, CSF2RB, GPER1, S100A12, PKN1, CD177, BMP6, KYNU, CEACAM1, KCNN4, SPNS2, RAC2, PLA2G2F, SLA, RAPGEF3, IL1B, SERPINB4, ARG1, CTSS, CX3CL1, IL36RN, LAT2, TNFSF14, CD68, NLRP10, GBP5, SERPINB9, SLAMF9, IL36B, PI3, SMAD6,CXCL6, SRMS, GPRC5B
GOBP_BIOLOGICAL_ PROCESS_INVOLVED IN_INTERSPECIES_ INTERACTION BETWEEN_ORGANISMS	TMPRSS2, SH2D1B, REN, IL36A, DEFB4A, ITGAX, PLAAT3, CSF3, VTCN1, LCE3C, LTF, CXCR4, OASL, LCN2, MEFV, WFDC2, NCF2, RNASE7, IL23A, LCE3A, CFB, NRROS, TH, VNN1, IL36G, A2M, CSF2RB, GPER1, S100A12, CD177, BMP6, KYNU, CEACAM1,PLA2G2F, SLA, IL1B, SERPINB4, ARG1, DUOX2, GSDMA,TIMP4, CX3CL1, AKAP12, IL36RN, CD68, NLRP10, GBP5, SERPINB9, IL36B, PI3, SMAD6, CXCL6, SERPINB3, SRMS
GOBP_DEFENSE_ RESPONSE	SH2D1B, DUOXA2, IL36A, DEFB4A, ITGAX, SAA1, IL1RL1, SERPINA1, LCE3C, LTF, CXCR4, ALOX5, ITGB2, SAA2, OASL, LCN2, MEFV, WFDC2, NCF2, RNASE7, IL23A, LCE3A, CFB ,NRROS, NFAM1, VNN1, IL36G, A2M, GPER1, S100A12, CD177, BMP6, KYNU, LYPD1, CEACAM1, CHST2, KCNN4, GAL, PLA2G2F, SLA, CAMK2N1, IL1B, SERPINB4, ARG1, DUOX2, GSDMA, CX3CL1, IL36RN, MMP3, CD68, NLRP10, GBP5, SERPINB9
GOBP_INNATE_ IMMUNE_RESPONSE	SH2D1B, IL36A, LTF, OASL, LCN2, MEFV, WFDC2, NCF2, RNASE7, IL23A, CFB, NRROS, VNN1, IL36G, A2M, GPER1, S100A12, CD177, KYNU, CEACAM1, PLA2G2F, SLA, SERPINB4, ARG1, CX3CL1, IL36RN, NLRP10, GBP5, SERPINB9, IL36B, PI3, SRMS
GOBP_EPITHELIAL_ CELL_DIFFERENTIATION	SPRR2F, ROBO4, PLAAT3, LCE3C, CXCR4, PLAAT4, SLC44A4, LCE3E, XDH, SPRR3, KRT13, SPRR2A, DHRS9, LCE3D, STC1, LCE3A, KRT19, SPRR2B, FOXA1, SPRR2E, AGR2, RHCG, SPRR2D, BMP6, SPRR2G, CEACAM1, RAPGEF3, IL1B, CNFN, TJP3, DAB2, HEY1, TGM1, KRT23, ID3, ALOX15B, ATOH8, CDH2, TIE1,DLX3, LAMA1, PDE2A, SULT1B1, CDSN, CES1, LCE1F, HPSE, POF1B
GOBP_INFLAMMATORY RESPONSE	DUOXA2, IL36A, SAA1, IL1RL1, SERPINA1, CXCR4, ALOX5, ITGB2, SAA2, MEFV, IL23A, NRROS, NFAM1, VNN1, IL36G, A2M, GPER1, S100A12, BMP6, CHST2, GAL, CAMK2N1, IL1B, CX3CL1, IL36RN, MMP3, CD68, NLRP10, GBP5, IL36B, CXCL6, GPRC5B, C3, CXCL5,GBA, ADGRE5
GOBP_CENTRAL_NERVOUS_ SYSTEM_DEVELOPMENT	MAPT, GJC2, RECK, SOX6, SLIT3, HOXB3, CTNNA2, WNT4, S1PR1, FOXP2, ROBO2, NHLH2, LOXL3, ASPA, PCSK1, GLI2, SCN1B, SELENOP, PCDH18, PLXDC1, SLC8A1, SHROOM4, CLU

Pathways Dermis	Leading edge genes
GOBP_CYTOKINE_MEDIATED SIGNALING_PATHWAY	MMP12, HCK, IL13RA2, CRLF2, MT3, CCL5, CXCR4, CXCL8, CCL3, HSPA1B, HSPA1A, LEP, CCL26, CCL19, IL36B
GOBP_REGULATION_OF_RESPONSE TO_EXTERNAL_STIMULUS	MAS1, MMP12, HCK, MT3, CCL5, CXCR4, ADORA2A, CXCL8, SERPINB2, MMP3, FGF18, CCL3, LEP, TNFSF14, CCL26, CCL19, MMP8, PDE2A, SEMA4D, NINJ1, SERPINF2, PTGES, TNFAIP6, TNFRSF1B, SIRPA
GOMF_CYTOKINE_RECEPTOR BINDING	CRLF2, CCL5, CXCL8, CCL3, TNFSF14, CCL26, CCL19, IL36B
KEGG_CHEMOKINE_SIGNALING PATHWAY	HCK, CCL5, CXCR4, CXCL8, CCL3, CCL26, CCL19
GOBP_POSITIVE_REGULATION_OF IMMUNE_SYSTEM_PROCESS	MMP12, HCK, IL13RA2, CRLF2, CCL5, CXCL8, CCL3, CD247, LEP, TNFSF14, CCL19, IL36B, IGFBP2, BDKRB1, VNN1,MMP8, SIRPB1, GATA2
GOBP_RESPONSE_TO_CYTOKINE	MMP12, HCK, IL13RA2, CRLF2, MT3, CCL5, CXCR4, CXCL8, CCL3, HSPA1B, HSPA1A, LEP, INA, CCL26, CCL19, IL36B, SLC2A4, PDE2A, GCH1, RBM15, TNFRSF1B, RAB20, SIRPA, FZD4
GOBP_INFLAMMATORY_RESPONSE	MAS1, HCK, CCL5, CXCR4, ADORA2A, CXCL8, MMP3, CCL3, TREM1, LEP, CCL26, CCL19, IL36B, BDKRB1, VNN1, MMP8, PDE2A, NINJ1, SERPINF2, PTGES, TNFAIP6, TNFRSF1B, SIRPA
GOBP_DEFENSE_RESPONSE	MAS1, MMP12, HCK, CCL5, CXCR4, ADORA2A, CXCL8, MMP3, CCL3, ASIC4, TREM1, LEP, CCL26, CCL19, IL36B, PRG2, BDKRB1, VNN1, MMP8, PDE2A, GCH1, NINJ1, SERPINF2, PTGES, TNFAIP6, WFDC2, TNFRSF1B, RAB20, SIRPA
GOMF_EXTRACELLULAR_MATRIX STRUCTURAL_CONSTITUENT CONFERRING_TENSILE_STRENGTH	COL24A1, COL14A1, COL15A1, COL4A5, COL6A5, COL21A1, COL6A6
GOCC_COLLAGEN_TRIMER	COL24A1, COLEC12, COL14A1, C1QTNF2, COL15A1, COL4A5, COL6A5, COL21A1, COL6A6
GOCC_COLLAGEN_CONTAINING EXTRACELLULAR_MATRIX	POSTN, VCAN, EDIL3, FN1, ANXA2, COL24A1, ANGPT1, COL14A1, COL15A1, COL4A5, FGFR2, THSD4, COL6A5, ASPN, INHBE, COL21A1, SMOC2, COL6A6,OGN, F13A1, OMD
GOBP_CELL_CELL_ADHESION VIA_PLASMA_MEMBRANE ADHESION_MOLECULES	NEXN, PCDHA3, PCDHA10, PCDHAC1, PCDHA6, PCDHA7, PCDHA4, PCDHA11, PCDHA12, PCDHA8, PCDHA9, PCDHA1, PCDHA2, PCDHA5, PCDHA13, PCDHAC2, FLRT3

Fig S3: Leading genes upon NLRP1 activation. Leading edge genes are those that contribute most to the enrichment of a specific gene set within a pathway. The table shows leading genes identified by GSEA of NLRP1 activation in control SEs and ASC knockout SEs compared to inflammatory pathways. The top table corresponds to the epidermis, and the bottom table to the dermis.

Abbreviation: GSEA, gene set enrichment analysis; SE, skin equivalent.

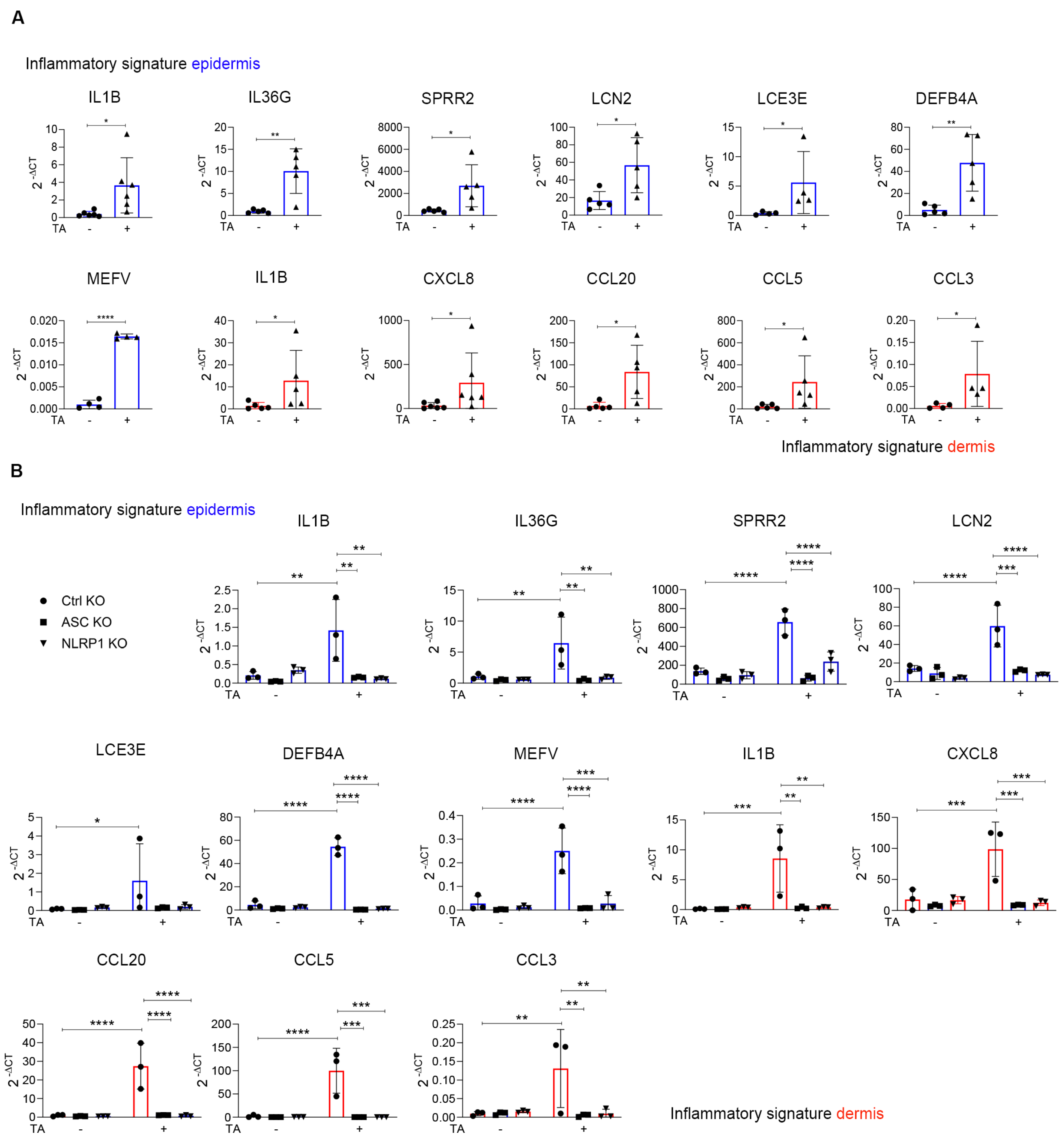

Fig. S4: The NLRP1-dependent upregulation of pro-inflammatory genes is donor-independent and ASC- and NLRP1- dependent. (A) SEs generated with HPKs from 5 different donors were mock-treated or treated with talabostat (0.3 μM) for 3 days. Expression of the

indicated genes by keratinocytes (blue) or fibroblasts (red) was analyzed by qPCR and normalized to HPRT expression. Data represent the average between 2 replicates from ≥ 4 different donors. P values were calculated with one-tailed unpaired t-test. (∗∗∗∗P < 0.0001, ∗∗∗P ≤ 0.001, ∗∗P ≤ 0.01, and ∗P ≤ 0.05, ns = not significant). **(B)** SEs generated with control, ASC, or NLRP1 knockout HPKs were mock-treated or with talabostat (0.3 μM) for 3 days. Expression of the indicated genes by keratinocytes (blue) or fibroblasts (red) was analyzed by qPCR and normalized to HPRT expression. Data are represented by mean ± SD of 3 replicates. P values were calculated with two-ways ANOVA. (∗∗∗∗P < 0.0001, ∗∗∗P ≤ 0.001, ∗∗P ≤ 0.01, and ∗P ≤ 0.05, ns = not significant).

Abbreviation: TA, talabostat; SE, skin equivalent; HPKs, human primary keratinocytes.

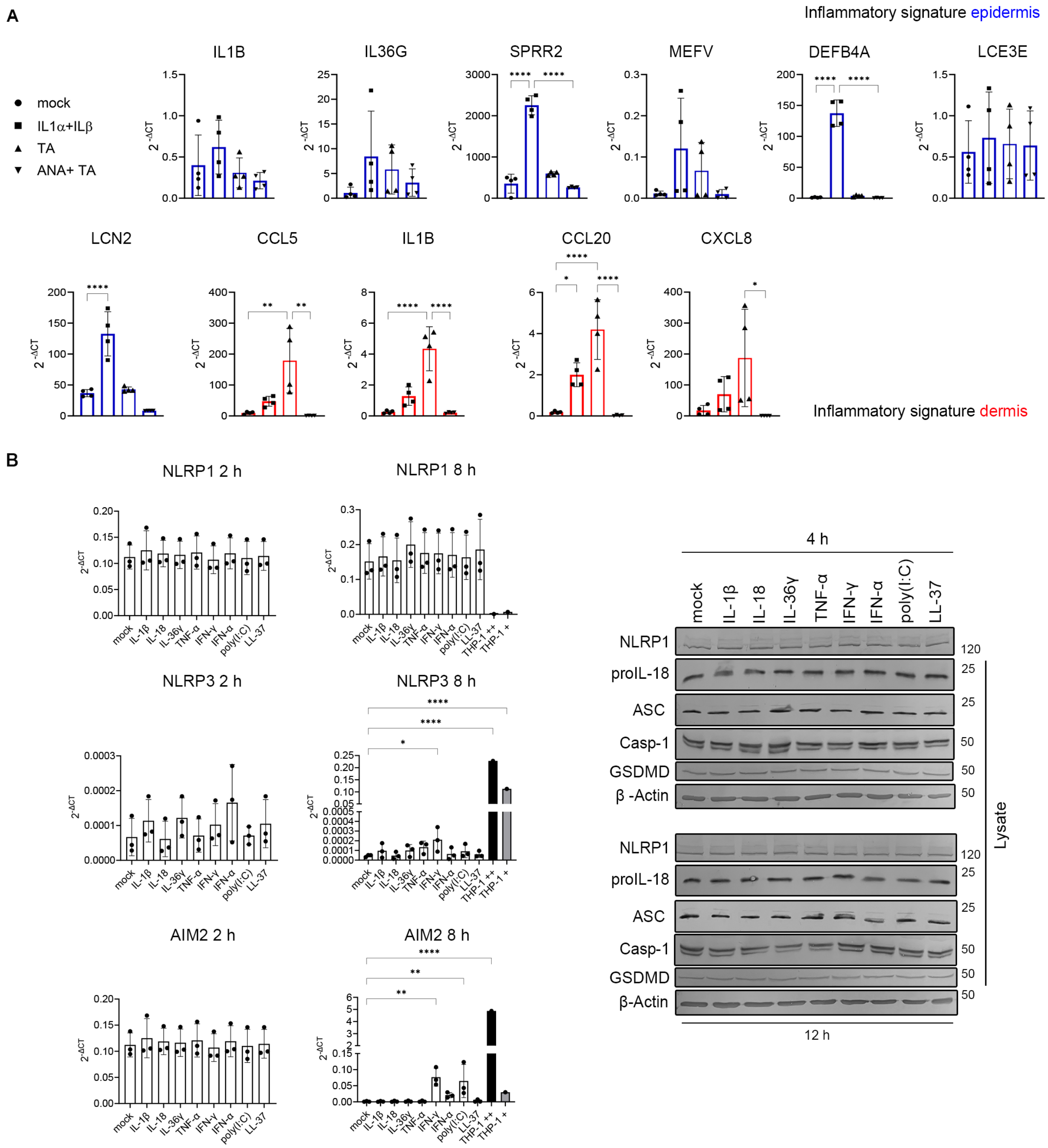

Fig. S5: IL-1 is the major effector of NLRP1 inflammasome activation.

(A) SEs were mock-treated or with IL-1α/β (1 ng/ml each), talabostat (0.3 μM), or anakinra (10 μg/ml) plus talabostat, for 1 day. Expression of mRNA of representative genes of the epidermis (blue) and of the dermis (red) was determined by qPCR and normalized to HPRT expression. **(B)**

HPKs from 3 different donors in monolayer were treated with IL-1β (10 ng/ml), IL-18 (20 ng/ml), IL-36γ (100 ng/ml), TNFα (10 ng/ml), IFN-γ (20 ng/ml), IFN-α (10 ng/ml), poly(I:C) (1 μg/ml), or LL-37 (1 μg/ml), and NLRP1, NLRP3 and AIM2 expression was determined at the RNA level 2 h and 8 h post stimulation (left) and inflammasome-related genes at the protein level after 4 h and 12 h (right). β-actin expression served as control. THP-1 + (PMA 50 ng/ml) and THP-1 ++ (PMA 50 ng/ml, LPS 0.1 μg/ml) are used as positive control for NLRP3 and AIM2 RNA expression. Data are represented by mean ± SD of **(A)** 4 replicates and are representative of 2 independent experiments or **(B)** 3 different donors, or a representative blot out of 3 is shown. P values were calculated with one-way ANOVA (∗∗∗∗P < 0.0001, ∗∗∗P ≤ 0.001, ∗∗P ≤ 0.01, and ∗P ≤ 0.05, ns = not significant).

Abbreviation: SE, skin equivalent; HPKs, human primary keratinocytes, TA, talabostat; ANA, anakinra.

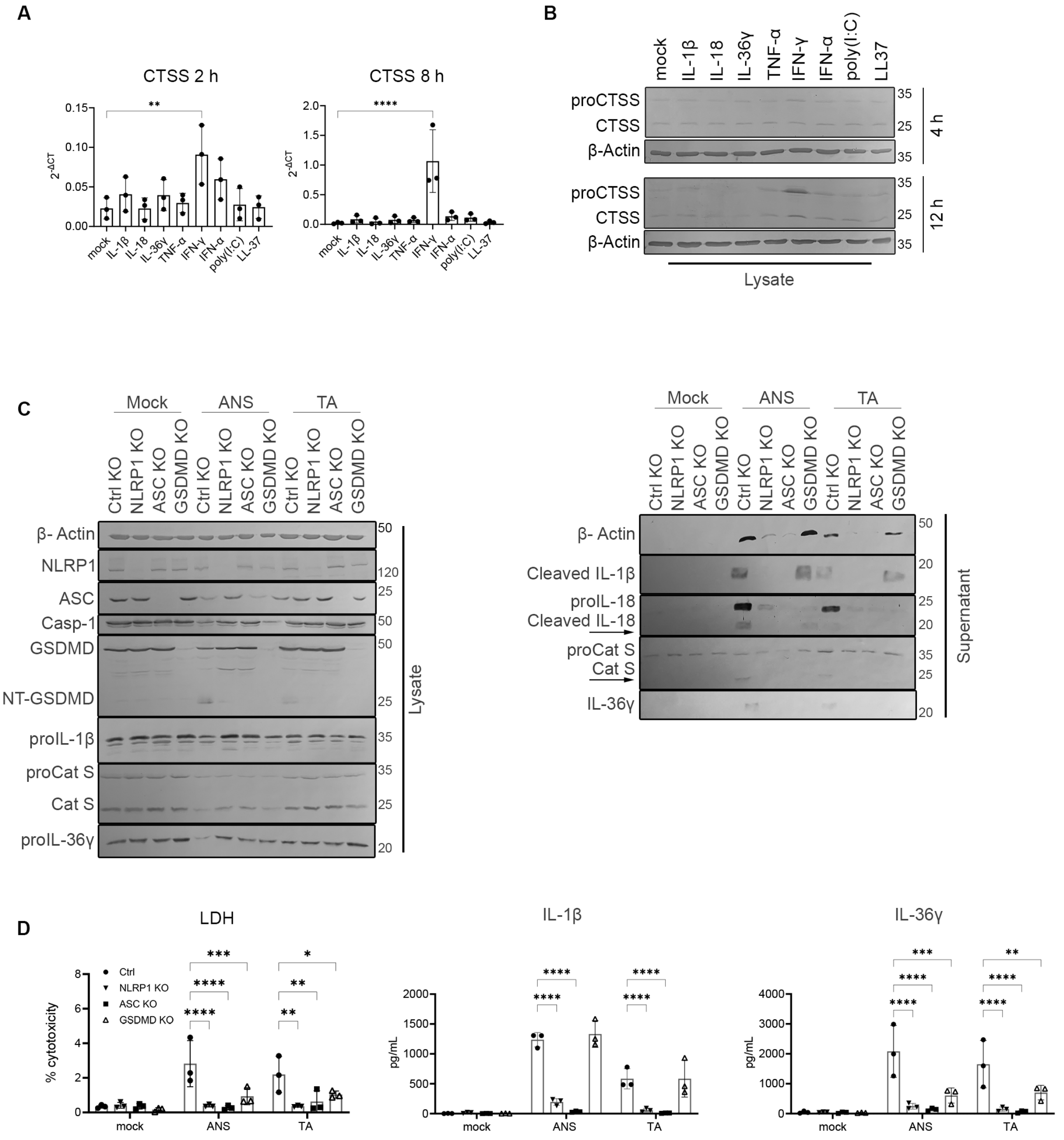

Fig. S6: Active cathepsin S is secreted upon NLRP1 inflammasome activation.

(A, B) HPKs from 3 different donors in monolayer were treated with IL-1β (10 ng/ml), IL-18 (20 ng/ml), IL-36γ (100 ng/ml), TNFα (10 ng/ml), IFN-γ (20 ng/ml), IFN-α (10 ng/ml), poly(I:C) (1 μg/ml), or LL-37 (1 μg/ml), and CTSS expression was determined at the **(A)** RNA level 2 h and 8

h post stimulation and at the **(B)** protein level after 4 h and 12 h. HPRT and β-actin expression were used as internal controls, respectively. **(C, D)** Polyclonal knockout HPKs from 3 different donors in 2D monolayer were primed with IL-1α (10 ng/ml) and IFN-γ (20 ng/ml) and mock-treated or with anisomycin (1 μM, 5 h), or talabostat (0.3 μM, 12 h). Protein expression and release was determined by **(C)** western blot or **(D)** ELISA. **(D)** Cell death was measured by LDH release. Data are represented by mean ± SD of 3 different donors or a representative blot out of 3 experiments is shown. P values were calculated by two-way ANOVA (∗∗∗∗$P < 0.0001$, ∗∗∗$P \leq 0.001$, ∗∗$P \leq 0.01$, and ∗$P \leq 0.05$, ns = not significant).

Abbreviation: HPKs, human primary keratinocytes, ANS: anisomycin; TA: talabostat.

Leading edge genes: Ctrl SE TA Epidermis vs psoriatic epidermis

BASAL2 UP	DEFB4A, CXCR4, LCN2, SPRR2A SPRR2E, CHI3L2, VNN1, IL36G, SPRR2D, KYNU, CHST2, RAC2, SLA, SERPINB4, S100A7A, CNFN, CD68, SLCO4A1, PI3,SERPINB3, ADIRF, OLFM1, LRG1, ALOX15B, DLX3, KRT75, CSTB
MITOTIC UP	DEFB4A, CXCR4, LCN2, SPRR2A, SPRR2E, CHI3L2, VNN1, IL36G,RHCG, CSTB, SPRR2D, KYNU, CHST2, RAC2, SLA, MUCL1, iL1B, SERPINB4, S100A7A, CNFN, CD68, PI3, SERPINB3, ADIRF, TGM1,OLFM1, LRG1, ALOX15B, DLX3, KRT75,
BASAL1 UP	DEFB4A, CXCR4, LCN2, SPRR2A,SPRR2E, CHI3L2, VNN1, IL36G, SPRR2D, KYNU, CHST2, SLA, IL1B, SERPINB4, S100A7A, CNFN, CD68, PI3, SERPINB3, ADIRF, TGM1, OLFM1, LRG1, ID3, ALOX15B, KRT75, CSTB
FOLLICULAR UP	DEFB4A, CXCR4, SPRR2A,SPRR2E, VNN1, IL36G, SPRR2D, RAC2, S100A7A, PI3
GRANULAR UP	DEFB4A, SPRR2A, IL36G, RHCG, SPRR2D, SERPINB4, S100A7A, CD68, TGM1, LRG1
CHANNEL UP	DEFB4A, SPRR2A, SPRR2E, CHI3L2, VNN1, IL36G, RHCG, SPRR2D, KYNU, SERPINB4, S100A7A, CNFN, PI3, SERPINB3, ADIRF
CHANNEL DOWN	HCFC1R1,CCND1,GLTP, PLIN2, CD24, SCCPDH, EFEMP1, SULF2, TNFRSF19, C12orf75, LURAP1L, WNT4, LAMB4, SYNPO2, RERG, KLK1
BASAL1 DOWN	GLTP, PLIN2, TMEM40, ZNF165, SPTLC2, PDGFC, WNT3, EFEMP1, SULF2, KITLG, CA2, TNFRSF19, RASSF9, SOX6, BBOX1, SYNE1, LAMB4, SYNPO2, LRRN4CL,RERG
MITOTIC DOWN	BACE2, CCND1, GLTP, PLIN2, TMEM40, GRB7, SPTLC2, PDGFC, WNT3, SCCPDH, EFEMP1, SULF2, TCEA3, CABLES1, KITLG, CA2, TNFRSF19, RASSF9, HNMT, RASSF6, PTGS1, SOX6, BBOX1, SYNE1, LAMB4, SYNPO2 ,TNFRSF18, LRRN4CL,RERG
BASAL2 DOWN	WNT3, GPR155, SCCPDH, EFEMP1, SULF2, TCEA3, CABLES1, KITLG, CA2, TNFRSF19, RASSF9, HNMT, RASSF6, PTGS1, LURAP1L, SOX6, BBOX1, SYNE1, LAMB4, SYNPO2, TNFRSF18, MYLK, LRRN4CL, RERG, CLU, ASPN

Leading edge genes: Ctrl SE TA Epidermis vs atopic dermatitis

ATOPIC_DERM KERA_UP	CA2, TNFRSF19, LURAP1L, LAMB4, ASPN

Fig. S7: Leading genes of regulated pathways

Table showing the leading-edge genes from GSEA of the epidermis of control SEs treated with talabostat compared to the epidermis of psoriatic or atopic dermatitis lesions.

Abbreviation: GSEA, gene set enrichment analysis; SE, skin equivalent.

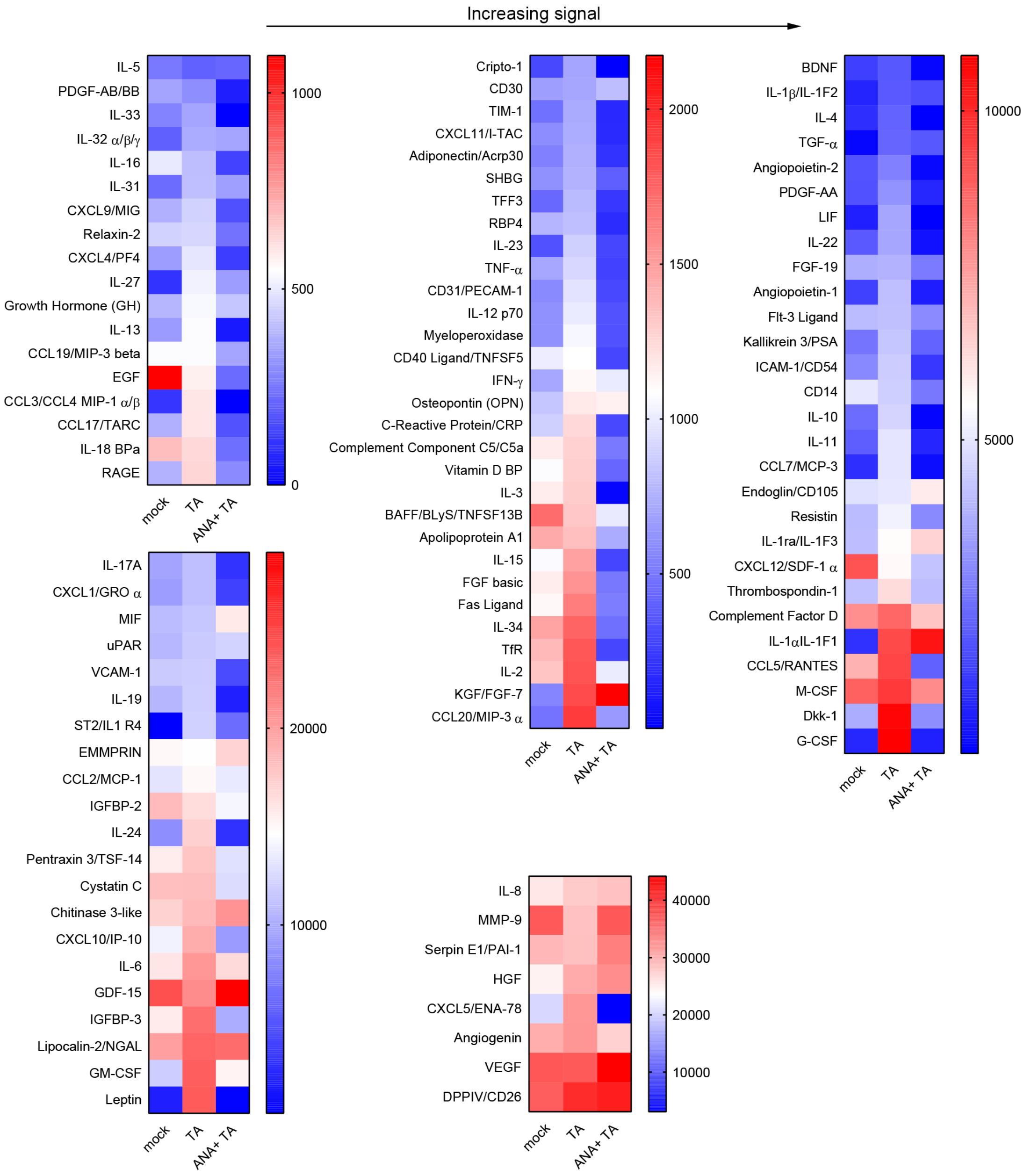

Fig. S8: NLRP1 activation regulates release of several inflammatory cytokines.

SEs with wild type HPKs were mock-treated or treated with talabostat (0.3 µM) or with anakinra (10 µg/ml) plus talabostat, for 3 days. Release of the indicated proteins was determined by cytokine array in a semi-quantitative manner.

Abbreviation: SE, skin equivalent; HPKs, human primary keratinocytes.

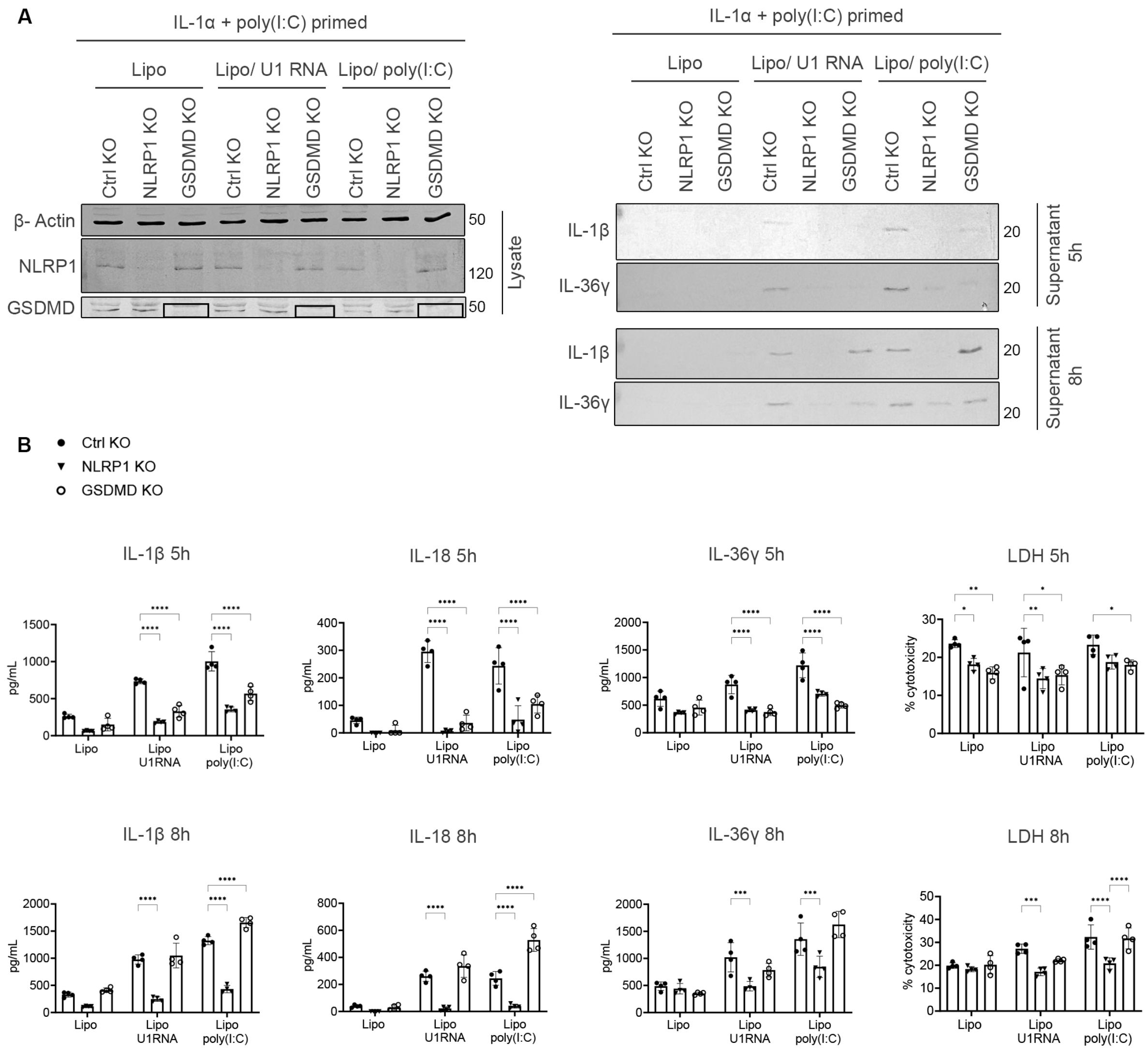

Fig. S9: U1 RNA induces IL-36γ release dependent on GSDMD pores.

(A,B) HPKs were primed overnight with IL-1α (10 ng/mL) and poly(I:C) (1 µg/ml) and then transfected with lipofectamine (Lipo), Lipo/U1 RNA (1 µg/ml) or Lipo/poly(I:C) (1 µg/ml), for 5 h or

8 h. Protein expression and release was determined by **(A)** western blot or **(B)** ELISA. Cell death was measured by LDH release. **(A)** Blots are representative of two technical replicates. **(B)** Data are represented by mean ± SD of 4 technical replicates. P values were calculated by two-way ANOVA (∗∗∗∗P < 0.0001, ∗∗∗P ≤ 0.001, ∗∗P ≤ 0.01, and ∗P ≤ 0.05, ns = not significant).

Supplementary tables Materials and Methods

Supplementary table 1

Single-stranded DNA sequences for lentiviral transduction

sgRNA	Sequence 5'-3'
Non-targeting control (Ctrl)	Forward CACCGGTAGCGAACGTGTCCGGCGT<br>Reverse AAACACGCCGGACACGTTCGCTACG
ASC	Forward CACCGTAGAAGCTGACCAGCTTGT<br>Reverse AAACACAAGCTGGTCAGCTTCTAC
Caspase-1	Forward CACCGATTGACTCCGTTATTCCGAA<br>Reverse AAACTTCGGAATAACGGAGTCAATC
NLRP1	Forward CACCGCTCAGCCAGAGAAGACGAG<br>Reverse AAACCTCGTCTTCTCTGGCTGAGC

sgRNA sequences for electroporation

sgRNA	Sequence 5'-3'
Non-targeting control (Ctrl)	AAATGTGAGATCAGAGTAAT
ASC	CGCTAACGTGCTGCGCGACA
Caspase-1	TCCACTAGCATCTTACCTTG
NLRP1	CTGGATCCATGAATTGCCGG

Supplementary table 2

Real-time PCR primers

Primers	sequence 5'-3'
CCL3	Forward AGTTCTCTGCATCACTTGCTG<br>Reverse CGGCTTCGCTTGGTTAGGAA
CCL5	Forward AAGTTGTCTGTGTGCGCAAATCC<br>Reverse CCATTCCAGAAAAGCCACAGTTTT
CCL20	Forward CCAGCAGTCGTCTTT GTCAC<br>Reverse CTCTGGGTTGGCACACACTT
DEFB4A	Forward GGTGGTATAGGCGATCCTGTT<br>Reverse AGGGCAAAAGACTGGATGACA
HPRT	Forward ATTGTAATGACCAGTCAACAGGG<br>Reverse GCATTGTTTTGCCAGTGTCAA
IL-1B	Forward CACGATGCACCTGTACGATCA<br>Reverse GTTGCTCCATATCCTGTCCCT
IL36G	Forward AGGAAGGGCCGTCTATCAATC<br>Reverse CACTGTCACTTCGTGGAACTG
CXCL8	Forward TTTTGCCAAGGAGTGCTAAAGA<br>Reverse AACCCTCTGCACCCAGTTTTC
AIM2	Forward CAGAAATGATGTCGCAAAGCA<br>Reverse TCAGTACCATAACTGGCAAACAG
CASP-1	Forward TCCCTAGAAGAAGCTCAAAGGATATG<br>Reverse CGTGTGCGGCTTGACTTG
NLRP3	Forward GCAAAAAGAGATGAGCCGAAGT<br>Reverse GCTGTCTTCCTGGCATATCACA
CTSS	Forward GGATCACCACTGGCATCTCT<br>Reverse ATTCCCATTGAATGCTCCAG
SPRR2B	Forward ACGCCAAAGTGCCCAGAG<br>Reverse ATTTCTGCTGGCACTGCTGAG
LCN2	Forward CCACCTCAGACCTGATCCCA<br>Reverse CCCCTGGAATTGGTTGTCCTG

MEFV	Forward TAAGACCCCTAGTGACCATCTG<br>Reverse TTCCCCATAGTAGGTGACCAG
LCE3E	Forward AGTACAGTGTCTGCCTCCAGCT<br>Reverse CTGTCACAGGAGTTGGACCTCT

Supplementary table 3

Antibodies

**Antibody**	**Order number**	**Application**
β-actin	A5441 (Sigma)	WB
NLRP1	679802 (Biolegend)	WB
ASC	AL177 (Adipogen)	WB, IHC, IF
Caspase-1	sc-622 (Santa Cruz)	WB
Filaggrin	NBP1-87528	IHC
IL-18	PM014 (MBL)	WB
IL-1β	MAB201 (R&D)	WB
IL-36γ	AP2320 (R&D)	WB
CTSS	PA5-47088 (Thermofisher)	WB
GSDMDC1	NBP2-33422 (Novus)	WB
Isotype control mouse	ab37355 (abcam)	IHC, IF
Isotype control rabbit	ab172730 (abcam)	IHC, IF
Keratin-10	905404 (Biolegened)	IF
Keratin-15	GP-CK15	IF
Pan-cytokeratin	sc-8018	IF
SPRR2	AG-25B-0002	IHC
Anti-Rabbit IgG (Fc), AP Conjugate	s373b (Promega)	Secondary AB, WB
Anti-Mouse IgG (H+L), AP Conjugate	s372b (Promega)	Secondary AB, WB
Anti-Goat IgG, AP Conjugate	V115A (Promega)	Secondary AB, WB
Anti- Guinea pig IgG (H+L), AP Conjugate	ab6909 (Abcam)	Secondar AB, WB

Anti-mouse IgG (H+L) Alexa Fluor 488	A21429 (Life technologies)	Secondar AB, IF
Anti-mouse IgG (H+L) Alexa Fluor 647	A11001 (ThermoFisher)	Secondar AB, IF
Anti-mouse IgG BIOT	Ab5886	Secondar AB, IHC
Anti-Rabbit IgG BIOT	4010-08 (Southern Biotech)	Secondar AB, IHC

Supplementary Methods

Primary cell culture and treatment

Upon isolation, HPKs were cultured in keratinocyte serum-free medium (K-SFM, Thermo Fisher Scientific, MA, USA) supplemented with epidermal growth factor (EGF) and bovine pituitary extract (BPE). Cells were harvested using 0.05 % trypsin/0.02 % EDTA and cultured for ≥ 48 h before experimental use or for generation of skin equivalents.

Human dermal fibroblasts (HDFs) were isolated by incubating dermis in collagenase (1 mg/ml, Sigma) and 0.05 mM $CaCl_2$ in PBS for 2 h at 37°C. The partially digested dermis was mechanically dissociated in DMEM (high glucose, pyruvate, Thermo Fisher Scientific) containing 25% FBS (PAN-Biotech, Germany) and 1% antibiotic/antimycotic (Thermo Fisher Scientific). HDFs were expanded in DMEM with 10% FBS and 1% antibiotic/antimycotic and used to generate the fibroblasts-derived matrix-based dermal equivalents.

For 2D stimulations HPKs were treated in K-SFM with the following reagents:

- talabostat (3 µM, Lucerna-chem, Switzerland)
- rhIL-1β (10 ng/mL, Peprotech, NJ, USA), rhIL-1α (10 ng/mL, Peprotech, NJ, USA), rhIL-18 (20 ng/mL, Thermo Fisher Scientific), rhIL-36γ (100 ng/ml, R&D, MN, USA), rhTNF-α (10 ng/ml, InvivoGen, CA, USA), rhIFN-γ (20 ng/ml, InvivoGen)

- LL-37 (1 µg/ml, InvivoGen)
- poly(I:C) (1 µg/ml, InvivoGen)
- UVB irradiation (86.4 mJ/cm^2, UV802L, Waldmann, Germany)

Corresponding volumes of DMSO or PBS served as control.

CRISPR/Cas9 knockout of HPKs by Lentivirus transduction

Guide RNAs were designed using Benchling (https://benchling.com) and cloned into pLentiCRISPR v2 (#52961, Addgene) (sgRNA sequencing in Supplementary Table 1). HEK293T cells were co-transfected with pLentiCRISPR v2-sgRNA, psPAX2 (#12260, Addgene) and pMD2.G (#12259, Addgene). The supernatant containing viral particles was collected after 48 h, filtered (0.45 µm) and used to transduce freshly isolated HPKs plated on antibiotic-resistant feeder cells. Selection was performed with puromycin (5 µg/ml, Thermo Fisher Scientifc). Knockout efficiency was verified by western blot. Lentivirus-transduced knockout HPKs were used for the generation of skin equivalents.

CRISPR/Cas9 knockout of HPKs by elecroporation (RNP Method)

Cas9-sgRNA ribonucleoprotein (RNP) complexes were generated by combining recombinant Cas9 protein (Thermo Fischer Scientific) with custom-designed sgRNA (Benchling) at 1:1 molar ratio for 20 min at room temperature (RT). HPK pellets where resuspended in electroporation buffer R (5 µl buffer R per 0.15 × 10^6 cells) and mixed with RNPs. Electroporation was performed using 1700 V, 20 ms, 1 pulse. Cells were cultured for 1 week before experiments.

Generation of fibroblast-derived matrix dermal equivalents

For dermal equivalents, 5 x 10^5 HDFs were seeded onto 12-well ThinCerts (0.4 µm pores, 11.31 cm^2 culture area, VWR, PA, USA) on days 1, 3, and 5 (total 1.5 x 10^6 cells per insert) and cultured

for 3 weeks at 37°C, 5% CO_2, 20% O_2. Medium consisted of 3:1 DMEM/Ham's F12 (FAD medium) (Thermo Fisher Scientific) supplemented with 10% FBS (Sigma, Germany) and 1% antibiotic/antimycotic (Thermo Fisher Scientific), 2-phospho-l-ascorbic acid (200 µg/ml, Sigma) and the additional recombinant human proteins: TGF-β1 (1 ng/ml, Thermo Fisher Scientific), EGF (2.5 ng/ml, Thermo Fisher Scientific), bFGF (5 ng/ml, Peprotech), and insulin (5 µg/ml, Sigma). Medium was changed every 2 days over 4 weeks.

Generation of fibroblast-derived matrix skin equivalents (SEs)

To establish full-thickness skin equivalents, HPKs (2.5 x 10^5), either WT or CRISPR/Cas9-modified, were seeded onto dermal equivalents in FAD medium containing 10% FBS (Sigma) and 1% antibiotics/antimycotics (Thermo Fisher Scientific), supplemented with 2-phospho-l-ascorbic acid (200 µg/ml, Sigma), hydrocortisone (0.4 µg/ml, Sigma), and cholera toxin (1×10^{-10} M, Sigma). After 3 days submerged, SEs were air-lifted and cultured for 2 weeks with medium changes every other day.

Treatment of skin equivalents

Ten days post air-lift, SEs were stimulated with:

- talabostat (0.3 µM, Lucerna-Chem)
- anakinra (10 µg/ml, Swedish orphan biovitrum, Sweden)
- rhIL-1β or rhIL-1α (1 ng/ml, Peprotech)

Transfection of keratinocytes

HPKs in monolayer were transfected using Lipofectamine 2000 (Thermo Fisher Scientific). For each well, 1 µg of RNA (U1 (synthesized by Pascolo's group) or poly(I:C) (InvivoGen) was diluted

in 100 µl of K-SFM (Thermo Fisher Scientific). Separately, 2 µl of Lipofectamine 2000 (Thermo Fisher Scientific) were diluted in 100 µl of K-SFM (Thermo Fisher Scientific) and incubated 5 min. The solutions were combined and incubated for 15 min before being added dropwise to cells in 800 µl medium. Cells were incubated for 8 h at 37°C/5% CO_2.

RNA isolation and real-time PCR

Skin equivalents were homogenized using the TissueLyser II (3 min, 30 Hz, Qiagen, The Netherlands). Total RNA from monolayer or skin equivalents was isolated with Trizol (Qiagen) and retrotranscribed to cDNA. Levels of mRNA were determined by quantitative real-time PCR using the LightCycler 480 instrument and FastStart Essential DNA Green Master (Roche, Switzerland) and specific primers (Microsynth, Switzerland). mRNA levels were normalized to HPRT and RPL27. Primers can be found in Suppementay Table 2.

Histology and immunostaining

Samples were fixed in 4% formalin overnight, paraffin-embedded, sectioned at 5 µm, and stained with H&E.

Immunohistochemistry: tissue sections were deparaffinized in xylen, rehydrated in decresing concentrations of ethanol, and enogenous peroxidase was blocked with 3% H_2O_2 (Merck, Germany). Antigen retrival was performed using pH 9 Dako solution or citrate buffer (pH 6). Sections were blocked with 5% BSA in PBST (0.05% Tween 20 in PBS) and incubated with primary antibodies overnight at 4°C, followed by biotinylated secondary antibodies (1 h, room temperature) and detection with Vectastain Elite ABC HRP (Vector Laboratories, CA, USA) and AEC substrate (Dako, Denmark). Nuclei were stained using Mayer's hematoxylin (Kantonsapotheke, Switzerland). Slides were mounted with Faramount aqueous mounting medium (Dako) and scanned using an Aperio ScanScope (Leica Biosystems, Germany) or with

the PhenoImager Vectra Polaris (Akoya Biosciences, MA, USA). The primary and secondary antibodies used are specified in Supplementary Table 3.

Immunofluorescence: performed similarly but detected with fluorophore-conjugated secondary antibodies and counterstained with DAPI.

ASC speck quantification

Slides were scanned using PhenoImager Vetra Polaris (Akoya Biosciences). ASC specks were manually counted in QuPath (Queen's University Belfast, UK) and normalized to 100 μm section length.

ELISA and cytotoxicity assay

Cell culture supernatants were collected, cleared by centrifugation (400 g, 3 min) and release of human IL-1β, IL-1α, IL-18, IL-36γ or IL-8 was measured by ELISA (R&D Systems or Biolegend (IL-8)) according to manufacturer's instruction. Cytotoxicity was evaluated by measuring the release of lactate dehydrogenase (LDH) in the supernatant using the CytoTox 96 non-radioactive cytotoxicity assay (Promega, WI, USA). Total LDH was obtained by cell lysis in 10% Triton X-100 (Sigma). Results are shown as percentage release (LDH in SN/total LDH).

Proteome profiler human XL cytokine array

Cytokine array was performed using the supernatant from mock-, talabostat-, or anakinra plus talabostat-treated SEs using the proteome profiler human XL cytokine array kit (R&D Systems).

RNA-seq sample preparation

Three replicates of Ctrl SEs and ASC KO SEs were treated with talabostat (3 days). Epidermis and dermis were separated by dispase II digestion, homogenized with the TissueLyser II (Qiagen) and RNA was isolated using RNeasy Plus Universal Mini Kit (Qiagen). Libraries were prepared with approximately 0.1–1 µg total RNA using Illumina TruSeq Stranded mRNA and sequenced on Novaseq 6000 (single-end 100bp, 25M reads/sample, Functional Genomics Center Zurich, FGCZ).

Identification of differentially expressed genes (DEGs)

For epidermis analysis, contrasts included ctrl untreated versus ctrl talabostat-treated, ctrl untreated versus ASC KO untreated, ctrl talabostat-treated vs ASC KO talabostat-treated, ASC KO-untreated versus ASC KO talabostat-treated. The same contrasts were analyzed for dermis. For significant talabostat-treated unique genes, we chose genes with an absolute fold change > 2 and adjusted p-value < 0.05. If these genes had an absolute fold change > 2 in any of these contrasts ctrl-untreated versus ASC KO-untreated, ctrl talabostat-treated versus ASC KO talabostat-treated, or ASC KO-untreated versus ASC KO talabostat-treated, they were excluded. Multiple testing correction was performed with the Benjamini-Hochberg procedure. The gene expression data generated in this study were deposited in the Gene Expression Omnibus (GEO) under the accession number GSE282206.

RNAscope In situ hybridization (RNAscope)

RNAscope Multiplex Fluorescent Reagent Kit v2 (Advanced Cell Diagnostics, CA, USA) was used following manufacturer instructions. Briefly, subsequent tissue sections were pre-treated with the appropriate reagents for protease digestion. Specific RNA probes designed for human-NLRP1, human-AIM2 or human-NLRP3 were hybridized to the subsequent tissue samples, and signal amplification was achieved through a series of hybridization steps involving multiple amplification

probes. Fluorescent detection was performed using Opal 520 (Akoya Bioscience). Sections were counterstained with DAPI to visualize cell nuclei. The stained sections were imaged and scanned using the PhenoImager Vectra Polaris (Akoya Bioscience).

RNA visualization with SYTO® RNASelect™

OCT-embedded skin samples were sectioned at 10 µm and fixed in cold methanol (-20°C, 10 min). Sections were incubated with 500 nM SYTO RNASelect (Thermo Fisher Scientific) for 20 min at 37°C, washed in PBS, counterstained with DAPI, and imaged with FITC-compatible fluoresence microscopy.

Western blot

Monolayer cell lysates were harvested with SDS loading buffer and protein amounts were estimated and normalized by β-actin band quantification after immunoblotting. Epidermis and dermis of the skin equivalents were collected separately upon dispase II digestion (30 minutes, 37 ˚C) and lysed in a buffer containing 4% SDS and 100 mM TrisHCl pH 7.6, with TissueLyser II (3 min, 30/s Hz, QIAGEN). Proteins in solution were then incubated at 95˚C for 5 minutes, sonicated and separated from the pellet upon centrifugation (16.000 g, 5 minutes). Protein concentration was measured with the BCA protein assay (PIERCE, USA). Before loading in the gel, 0.1 M DTT was added to the mix and proteins were diluted 1:5 in 5X loading buffer (100% glycerol + 1% bromophenol blue).

Cell culture supernatants were precipitated with 2.5 volumes of acetone (100% w/v, Sigma-Aldrich, USA) by overnight incubation at -20˚C followed by centrifugation for ca. 2 h (4,000g at 4˚C) and resuspended in SDS loading buffer. Loading amount was quantified based on the normalized expression of β-actin in the lysate. Proteins were separated by SDS PAGE and analyzed by immunoblotting. The primary and secondary antibodies used are specified in Supplementary Table 3.

Immunofluorescence staining of cells

Cells were seeded on circular glass coverslips (18 mm) and fixed with 3% paraformaldehyde for 30 min at room temperature (RT). Following fixation, cells were permeabilized with 0.2% Triton X-100 for 2 min at RT and washed three times with PBS. Non-specific binding was blocked by incubating the cells in 1% BSA in PBS for 30 min at RT. The primary antibody diluted in blocking solution was then applied and incubated overnight at 4°C. The following day, cells were washed and incubated with the appropriate secondary antibody for 2 h at RT. Nuclei were counterstained with DAPI, and coverslips were mounted face-down onto glass slides using mounting medium.
