# Supplementary figures and images for: NLRP1 inflammasome activation in skin equivalents reveals mechanistic insights into the roles of keratinocytes in psoriasis
Source: Cell Death Dis. 2026 May 30;17(1):670. doi: 10.1038/s41419-026-08908-6 (PMC13424314; doi:10.1038/s41419-026-08908-6)

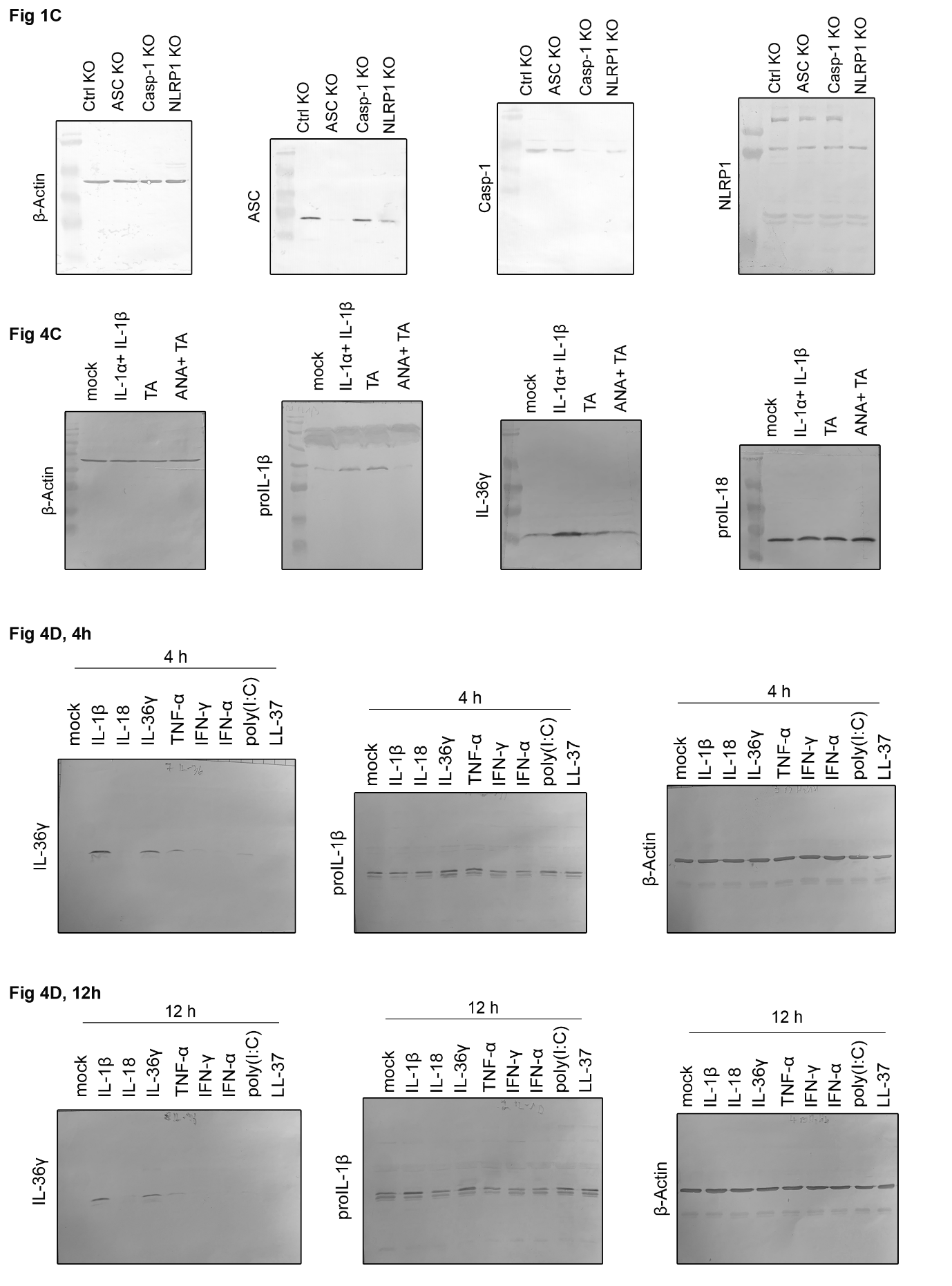


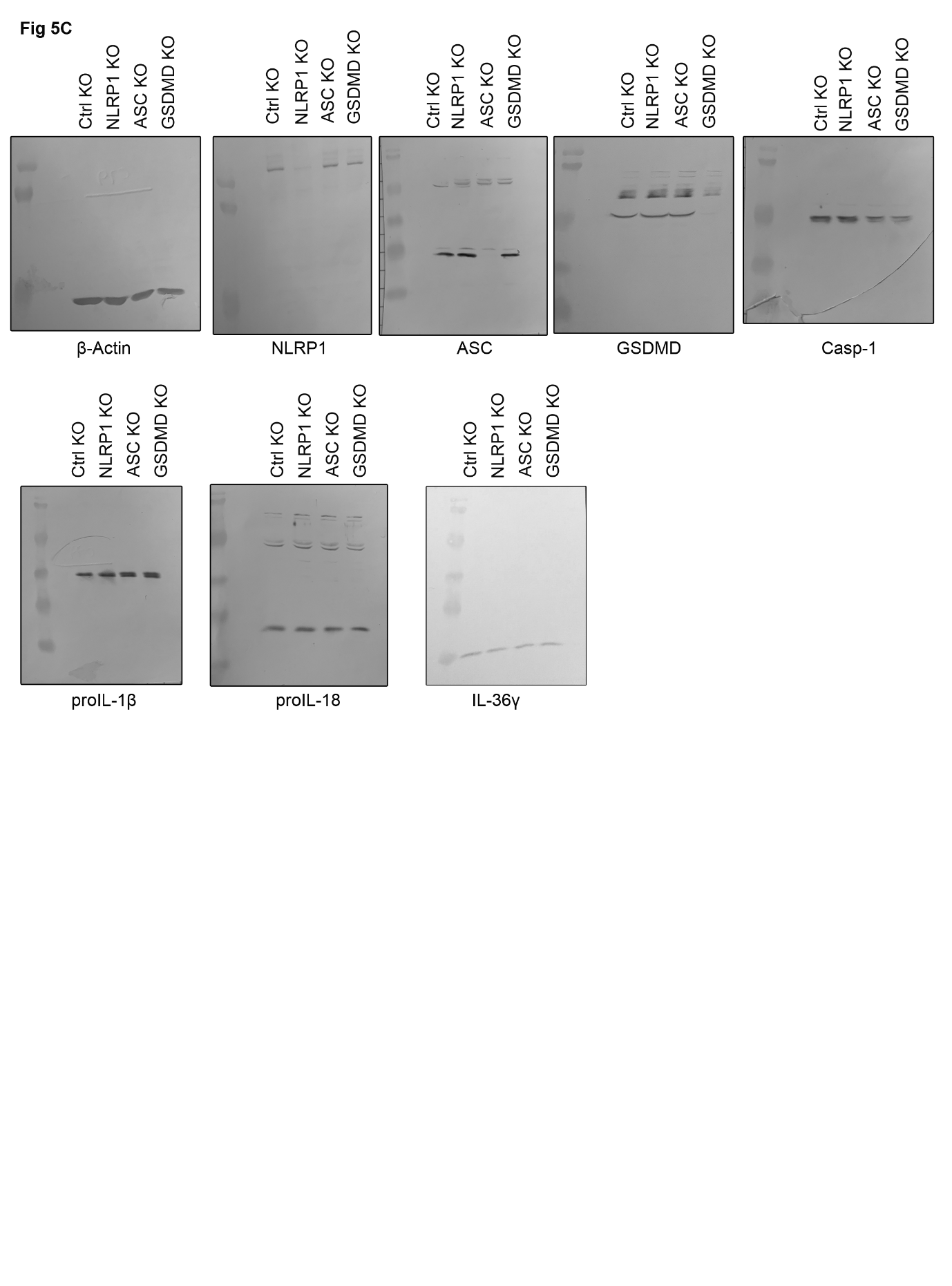


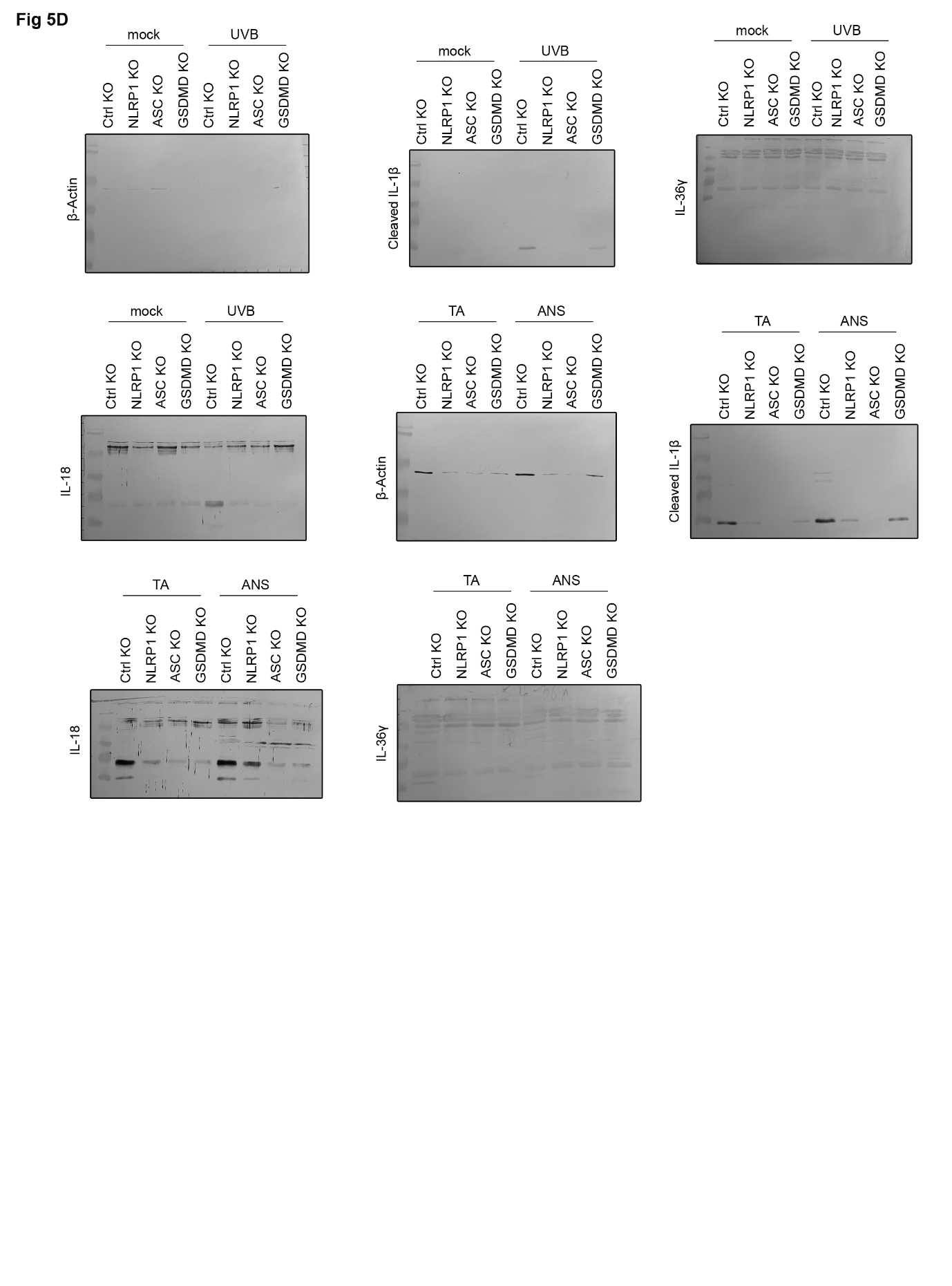


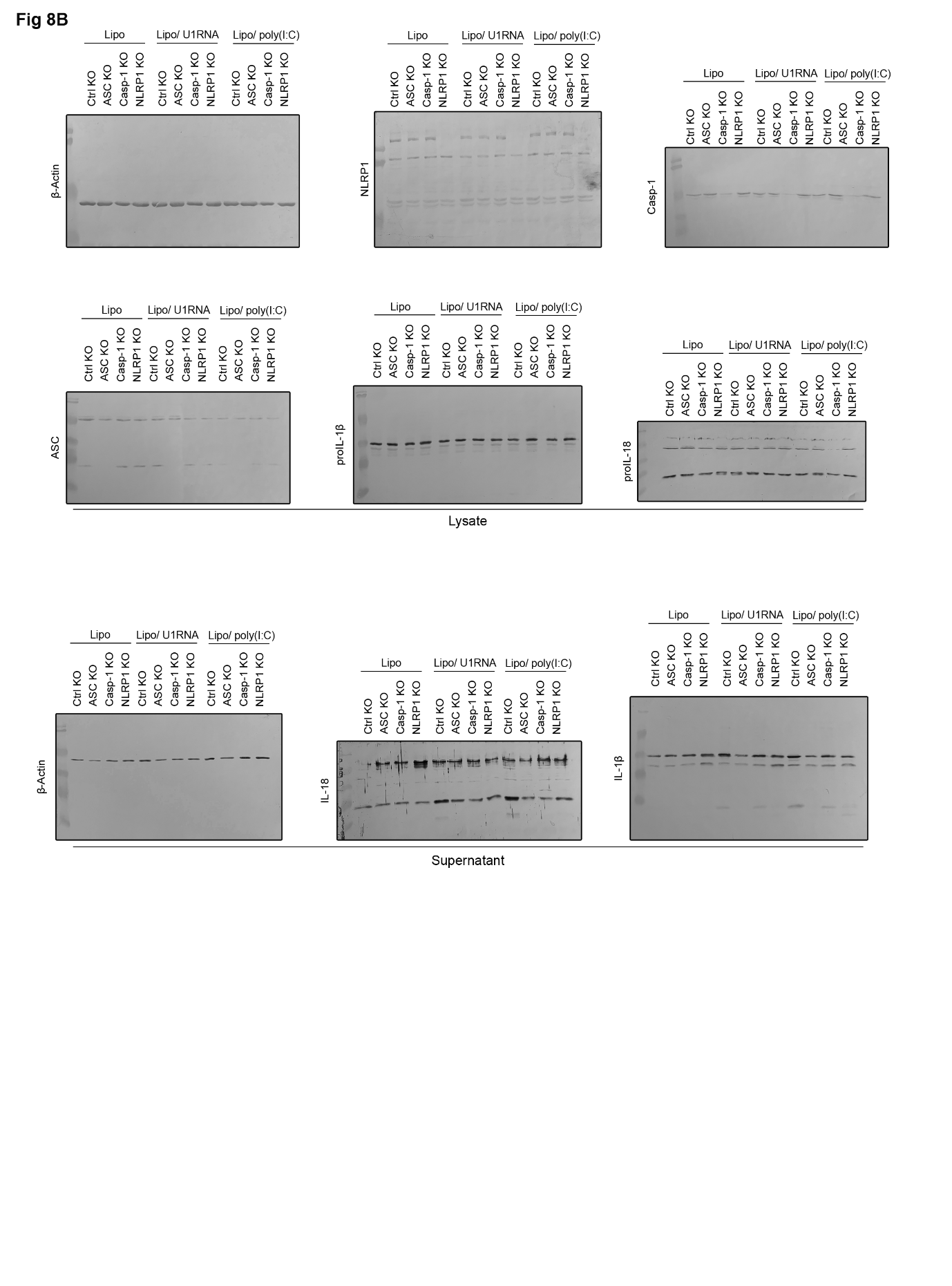


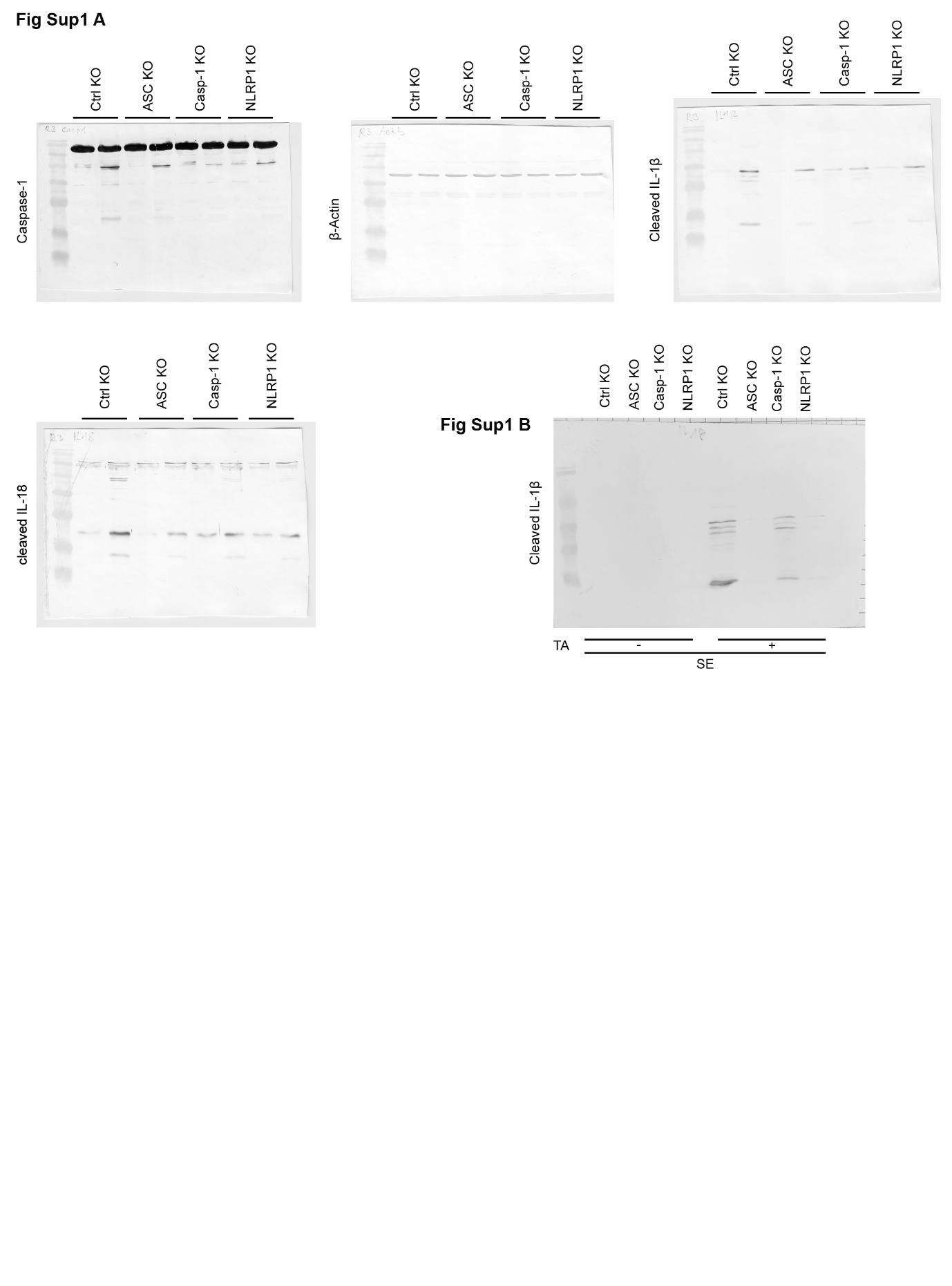


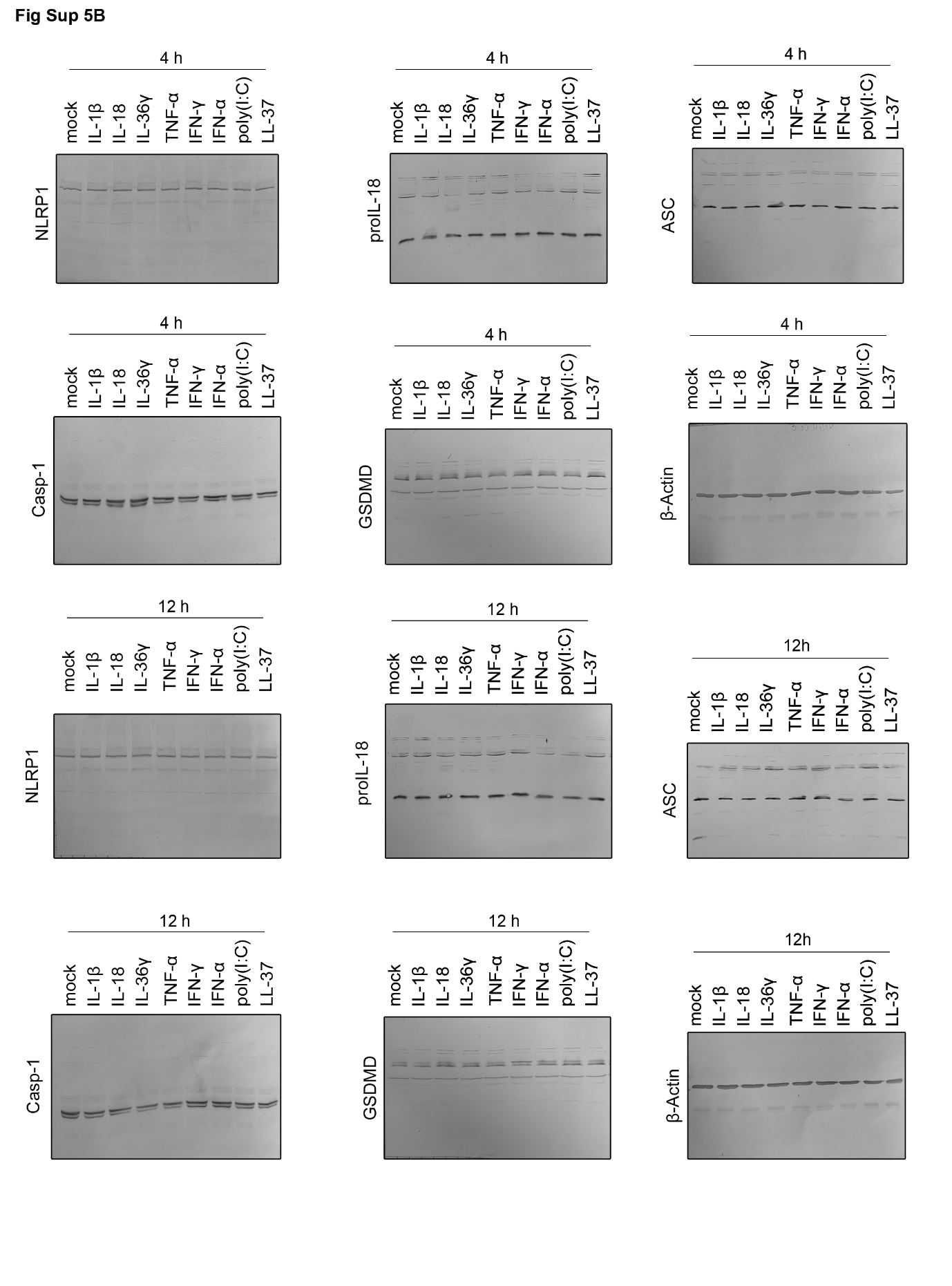


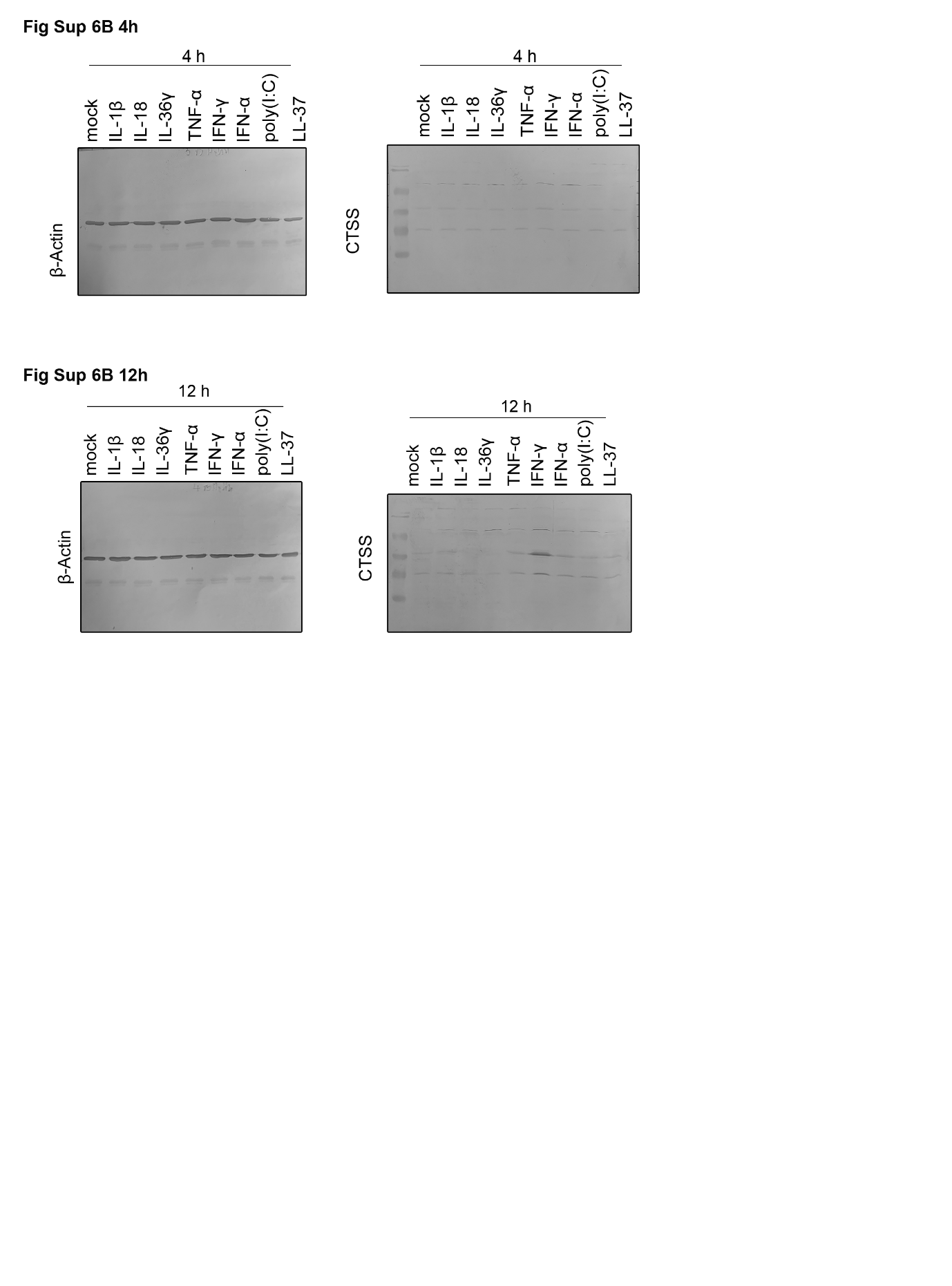


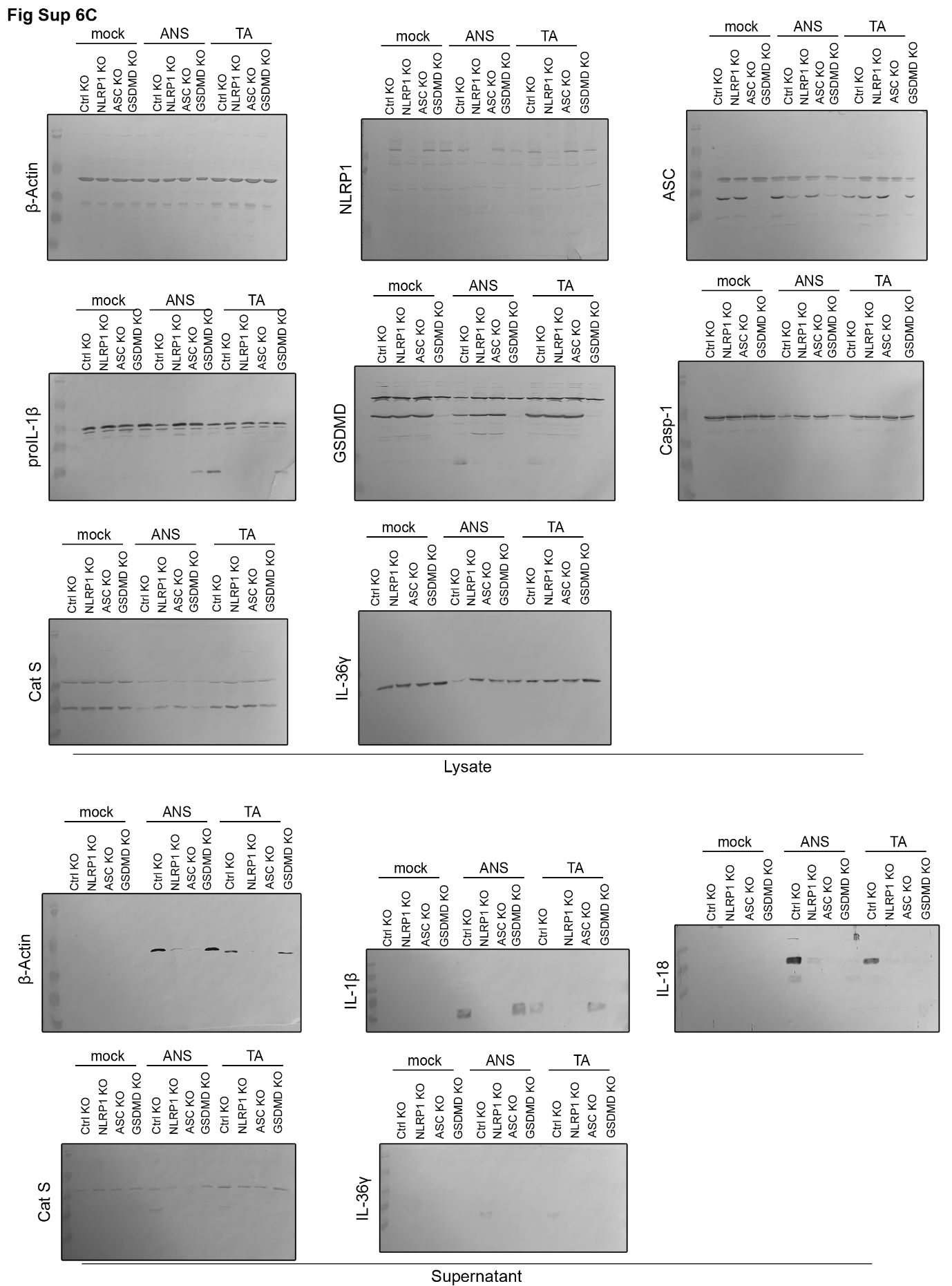


**
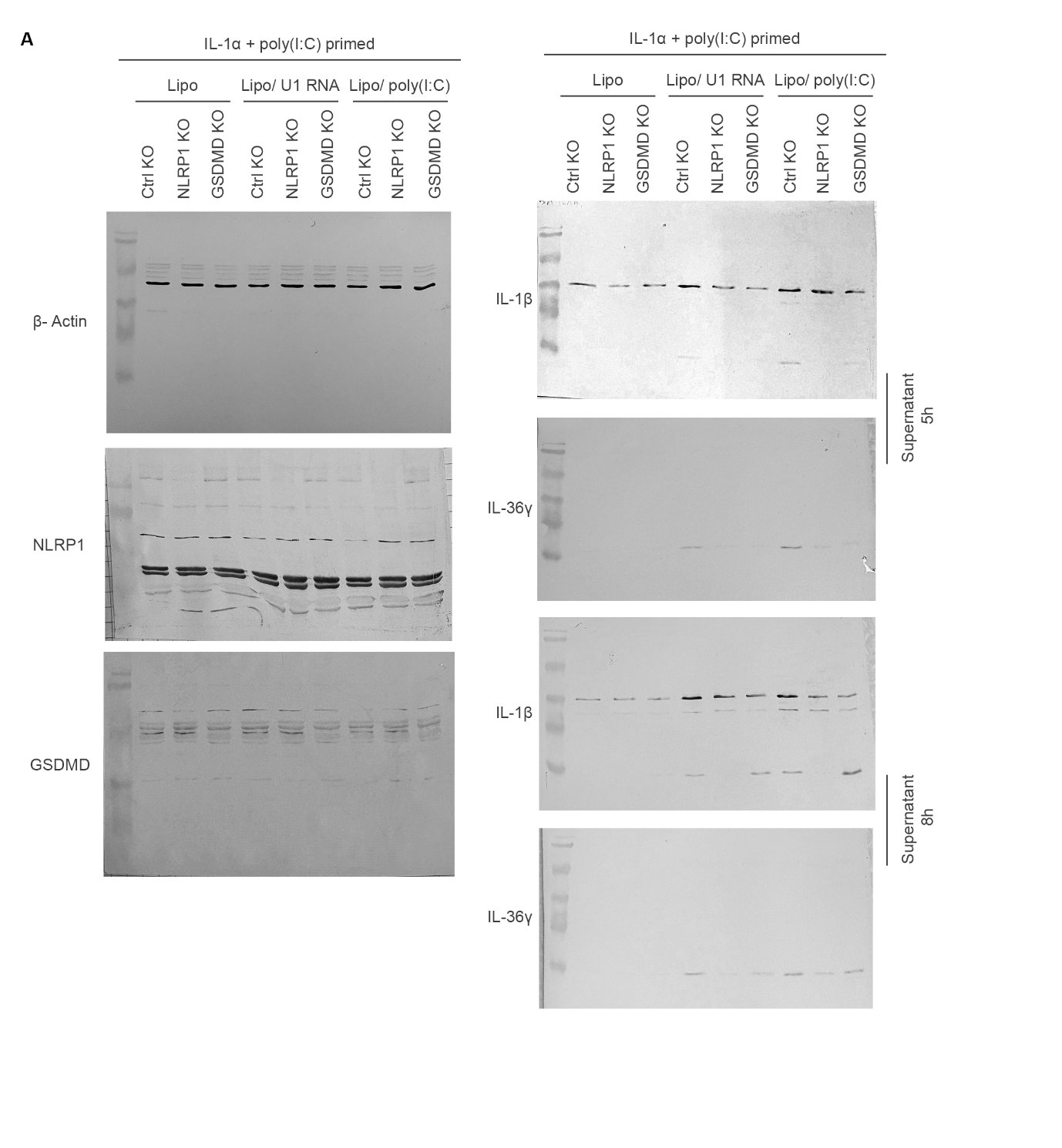
Fig Sup 9**

Supplement: Supplementary file 2 — Supplementary Methods [file 41419_2026_8908_MOESM2_ESM.docx]
